# Protecting cells by protecting their vulnerable lysosomes: Identification of a new mechanism for preserving lysosomal functional integrity upon oxidative stress

**DOI:** 10.1371/journal.pgen.1006603

**Published:** 2017-02-09

**Authors:** Raquel Pascua-Maestro, Sergio Diez-Hermano, Concepción Lillo, Maria D. Ganfornina, Diego Sanchez

**Affiliations:** 1 Instituto de Biología y Genética Molecular-Departamento de Bioquímica y Biología Molecular y Fisiología, Universidad de Valladolid-CSIC, Valladolid, Spain; 2 Instituto de Neurociencias de Castilla y León, Universidad de Salamanca, Salamanca, Spain; Stanford University School of Medicine, UNITED STATES

## Abstract

Environmental insults such as oxidative stress can damage cell membranes. Lysosomes are particularly sensitive to membrane permeabilization since their function depends on intraluminal acidic pH and requires stable membrane-dependent proton gradients. Among the catalog of oxidative stress-responsive genes is the Lipocalin Apolipoprotein D (ApoD), an extracellular lipid binding protein endowed with antioxidant capacity. Within the nervous system, cell types in the defense frontline, such as astrocytes, secrete ApoD to help neurons cope with the challenge. The protecting role of ApoD is known from cellular to organism level, and many of its downstream effects, including optimization of autophagy upon neurodegeneration, have been described. However, we still cannot assign a cellular mechanism to ApoD gene that explains how this protection is accomplished. Here we perform a comprehensive analysis of ApoD intracellular traffic and demonstrate its role in lysosomal pH homeostasis upon paraquat-induced oxidative stress. By combining single-lysosome *in vivo* pH measurements with immunodetection, we demonstrate that ApoD is endocytosed and targeted to a subset of vulnerable lysosomes in a stress-dependent manner. ApoD is functionally stable in this acidic environment, and its presence is sufficient and necessary for lysosomes to recover from oxidation-induced alkalinization, both in astrocytes and neurons. This function is accomplished by preventing lysosomal membrane permeabilization. Two lysosomal-dependent biological processes, myelin phagocytosis by astrocytes and optimization of neurodegeneration-triggered autophagy in a Drosophila *in vivo* model, require ApoD-related Lipocalins. Our results uncover a previously unknown biological function of ApoD, member of the finely regulated and evolutionary conserved gene family of extracellular Lipocalins. They set a lipoprotein-mediated regulation of lysosomal membrane integrity as a new mechanism at the hub of many cellular functions, critical for the outcome of a wide variety of neurodegenerative diseases. These results open therapeutic opportunities by providing a route of entry and a repair mechanism for lysosomes in pathological situations.

## Author summary

This work is the result of our search for the mechanism of action of Apolipoprotein D (ApoD), a neuroprotective lipid-binding protein that confers cell resistance to oxidative stress. ApoD is one of the few genes consistently over-expressed in the aging brain of all vertebrate species, and no nervous system disease has been found concurring without ApoD over-expression. All evidence supports ApoD as an endogenous mechanism of protection. We demonstrate here that this extracellular lipid binding protein is endocytosed and targeted in a finely controlled way to subsets of lysosomes in need of protection, those most sensitive to oxidative stress. ApoD reveals the existence of biologically relevant lysosomal heterogeneity that conditions the oxidation state of cells, their phagocytic or autophagic capacity, and the final output in neurodegenerative conditions. The stable presence of ApoD in lysosomes is sufficient and necessary for lysosomes to recover from oxidation-induced membrane permeabilization and loss of proton gradients. ApoD-mediated control of lysosomal membrane integrity represents a new cell-protection mechanism at the hub of many cellular functions, and is critical for the outcome of a wide variety of neurodegenerative diseases. Therapeutic opportunities open, by providing a route of entry and a repair mechanism for lysosomes in pathological situations.

## Introduction

Lysosomes are acidic intracellular vesicles that provide an optimal physicochemical milieu for enzymatic activities, most of them catabolic, which need to be controlled. A well-recognized lysosomal function is the degradation and recycling of defective cellular material through autophagy, and of extracellular material that reach lysosomes by endocytosis or phagocytosis. Newly documented functions such as energy and nutrient sensing, secretion, plasma membrane repair, immune response and cell death, reveal lysosomes as sophisticated organelles controlling fine decisions in the life of a cell [reviewed by 1,2,3].

Lysosome function is essential for human health, as clearly shown by the existence of numerous Lysosomal Storage Diseases [reviewed by 4], inherited metabolic disorders that affect a variety of tissues and organs and are particularly devastating for the nervous system. Moreover, lysosomal dysfunction is known to underlie the pathogenic mechanisms of neurodegenerative disorders, such as Alzheimer’s, Parkinson’s and Huntington’s diseases as well as physiological aging [5,6,7].

In the nervous system, proper lysosomal and autophagic functions are essential for the survival of postmitotic neurons, for the phagocytic activity of microglia and for the myelination process performed by Schwann cells and oligodendrocytes [8,9,10,11].

The stability of lysosomal membrane is a key factor determining cell survival-death signaling [12], and its composition is essential for an efficient lysosomal enzymatic activity [13]. Reactive oxygen species (ROS) compromise lysosomal integrity due to membrane lipid peroxidation [12,14]. Because of the critical role of its luminal pH, the alkalinization of lysosomes due to proton leakage is thought to contribute to many pathologies [15], and compensatory responses or therapeutic manipulations that restore lysosomal pH would result in clear benefits [16]. Interestingly, not all lysosomes are equally sensitive to oxidative stress or have the same luminal pH [14,17].

Apolipoprotein D (ApoD), a lipid binding protein of the Lipocalin family first known as part of bloodstream lipoprotein particles, is one of the few genes consistently over-expressed in the aging brain, and in all neurodegenerative and psychiatric diseases tested so far [reviewed in 18]. At the biochemical level, ApoD lipid binding properties are well known [19,20,21,22,23,24] and its ability to inhibit lipid peroxidation by reducing radical-propagating lipid hydroperoxides has been determined [25]. At the functional level, evidence for cellular protection and pro-survival roles for ApoD keep accumulating both, in animal models and cell systems. ApoD exhibits a functional pleiotropy, connecting nervous system response to oxidative stress [26,27,28,29,30], recovery after injury [31,32], brain aging [33,34,35], or diverse forms of neurodegeneration [36,37,38], as well as longevity regulation [39,40,41,42,43] or nervous system control of the metabolic adaptations to stress [44]. For instance, a lack of ApoD in mice compromise the process of myelin phagocytosis after peripheral nervous system injury [31,32], and the autophagic process was revealed as a key element in the degeneration rescuing activity of the ApoD *Drosophila melanogaster* homolog Glial Lazarillo (GLaz) [37]. How can an extracellular lipid binding protein accomplish these intracellular functions? ApoD is internalized by different cell types including astrocytes and neurons [26,28,45,46]. However, the ApoD-containing intracellular organelles or the dynamics and route of entry have not been identified. To achieve a mechanistic knowledge of ApoD neuroprotective actions, an in-depth analysis of its subcellular traffic is required.

In this work, we perform a comprehensive analysis of ApoD subcellular traffic and discover its role in lysosomal pH homeostasis and membrane stabilization under oxidative stress conditions. We demonstrate that ApoD is endocytosed and targeted to lysosomes in a stress-dependent manner. ApoD is functionally stable in this acidic environment, and actively helps to maintain lysosomal pH gradients both in astrocytes and in neurons, by maintaining lysosomal membrane integrity. Our analysis reveals ApoD as a specific marker for the subpopulation of lysosomes vulnerable to oxidation. We also test the notion that this lysosomal protecting mechanism is of wide biological consequences, by proving the role for this Lipocalin in two lysosomal-dependent biological processes such as myelin phagocytosis and optimization of neurodegeneration-triggered autophagy.

## Results

### Lipocalin rescue of polyglutamine-based neurodegeneration by autophagy optimization requires autophagosome-lysosome fusion

Using the Drosophila retina as a model system to assay neurodegeneration, we previously described that Type I Spinocerebellar Ataxia (SCA1) concurs with autophagic stress, showing an excessive or imbalanced induction of autophagy where autophagosome turnover is unable to keep pace with its formation [37]. GLaz, a Drosophila homologue of ApoD expressed by subsets of glial cells in the fly nervous system, has epistatic relationship with autophagy genes and optimizes clearance of aggregation-prone proteins such as the polyglutaminated form of human Ataxin 1 that is responsible for the SCA1 phenotype [37]. We concluded that GLaz rescues neurodegeneration by making autophagy more efficient, thus minimizing the negative effects of autophagic stress. We also proposed that the Lipocalin-mediated control of lipid peroxide levels influences autophagy at several steps, slowing down the process and ultimately making it more efficient. However, the mechanism for such an optimization of autophagy was not completely discerned.

**Fig 1:**
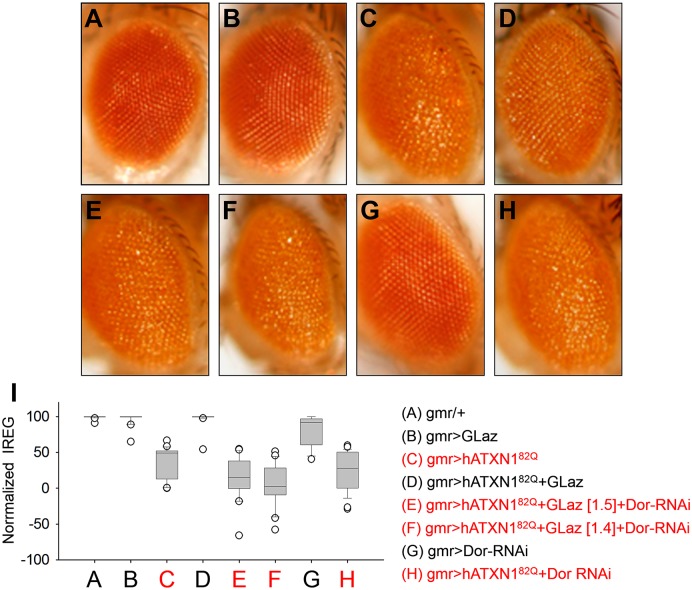
ApoD neuroprotection mechanism in polyglutamine-triggered neurodegeneration in Drosophila depends on lysosome-phagosome fusion *in vivo*. **A-H.** Representative examples of adult eye external morphology by light microscopy of the central eye surface region. The expression of all transgenes is directed by the gmr:Gal4 driver to photoreceptor neurons. Polyglutaminated human Ataxin 1 (UAS:hATXN1^82Q^) is combined with UAS:GLaz and/or UAS:Dor-RNAi transgenes. I. Quantification of photoreceptor degeneration by computing a regularity index (IREG) using FLEYE. No rescue is detected when Dor is knocked-down (confirmed with two independent recombinant lines, E and F). Data normalized to the control genotype (gmr/+) are shown. N = 20–30 flies/genotype. Statistical differences were assessed by ANOVA on Ranks and Tukey post-hoc correction. Genotypes C, E, F and H (degenerated) are significantly different from A, B, D, G (non-degenerated), p<0.001.

Here we evaluate retinal degeneration in the SCA1 fly retina model (Fig 1I) using FLEYE, a method for unbiased quantification based on the acquisition of fly eye surface pictures [47]. We combined the expression of polyglutaminated human Ataxin 1 (hATXN1^82Q^) with GLaz, and with DorRNAi, a knock-down of the HOPS complex subunit Vps18/Dor critical for tethering and fusing autophagosomes with lysosomes [48] (Fig 1). GLaz neurodegeneration rescue (Fig 1C and 1D, compare with controls in Fig 1A and 1B) is completely abolished by DorRNAi expression (Fig 1E and 1F). Dor down-regulation itself does not produce significant neurodegeneration in normal conditions (Fig 1G) nor modifies the neurodegeneration phenotype triggered by SCA1 in the fly retina (Fig 1H). These results demonstrate that the lysosome-autophagosome fusion event is required for GLaz optimization of autophagy that finally results in an efficient clearance of misfolded proteins in neurodegenerative conditions *in vivo*.

These results predict that Lipocalins must exert this function from the lysosome itself. Thus, a study showing the subcellular localization of ApoD-related Lipocalins once internalized in damaged neurons was required. To study the subcellular traffic of ApoD we switched to its native cell type, astrocytes, where we have reported its protective effect upon oxidative stress challenge, and its transcriptional regulation by the stress-responsive JNK pathway [26].

### Astroglial ApoD traffics through clathrin- and caveolin-dependent endocytic pathways, and concentrates prominently in the late endosomal-lysosomal compartment

We studied ApoD subcellular localization by detecting the native protein in astroglial cells (1321N1 astroglioma cell line), avoiding transfections that could alter its physiological traffic. A set of nine different markers for intracellular organelles were used to evaluate colocalization with ApoD in control, low serum (LS) and treatment with the oxidative stress-inducing agent paraquat (PQ) at 2 and 24 hours after stimulus.

**Fig 2:**
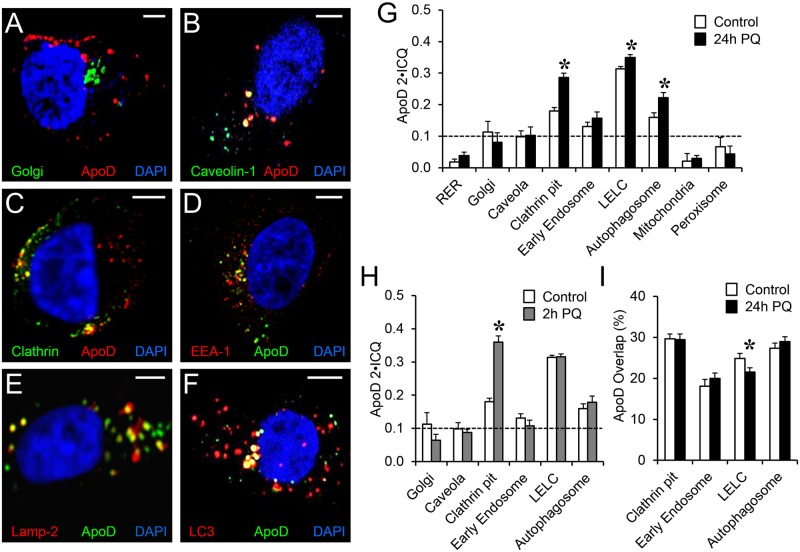
Astroglial ApoD traffics through clathrin- and caveolin-dependent endocytic pathways, concentrating in late endosomal-lysosomal compartment upon stress. **A-F.** Colocalization of ApoD with Golgi, caveola (Caveolin 1), Clathrin-coated pits and vesicles, early endosome compartment (EEA-1), late endosome-lysosome compartment (LELC, marked with Lamp-2), and autophagosomes (LC3) in 1321N1 cells. Representative sections of confocal microscopy z-stacks are shown. All markers were detected by immunocytochemistry, except for the Golgi apparatus, where cells are transfected with an organelle-directed GFP construct using the galactosyltransferase II signal sequence (see Methods). Colocalization appears in yellow. G-H. Average colocalization index referenced to ApoD signal (ApoD 2xICQ) for different 1321N1 organelles in control and after 24h paraquat (PQ) treatment (G), or after 2h PQ treatment (H). The dotted line represents the colocalization threshold (2xICQ<0.1 is considered due to chance). I. Spatial colocalization (Overlap referenced to ApoD signal) for the organelles showing ICQ-based index above threshold in G. Error bars in G-I represent SEM (n = 20 cells/marker from at least two independent experiments). Asterisks show statistical significance (p<0.05) assessed by Student’s t-Test between control and PQ conditions. Calibration bars in A-F: 5 μm.

Fig 2 and S1 Fig summarize the results of our image analysis from confocal z-stacks of at least 20 cells per condition, selected randomly from two independent experiments with triplicate wells (see Methods). Following two rounds of principal component analysis (PCA), we selected the intensity correlation quotient (ICQ) index [49] referenced to ApoD signal (ApoD ICQ) and the % Pixel Overlap referenced to ApoD signal (ApoD Overlap) to quantify ApoD protein targeted to each organelle (S1 Fig) (see Methods). We use a 2xICQ threshold of 0.1 for a colocalization not to be considered due to chance [50]. Since most variables covariate between control and LS, only PQ referred to the control condition is shown for simplicity in most figures.

ApoD concentrates significantly in Clathrin, EEA1, Lamp-2 and LC3-positive organelles, with particular prominence in the late endosome-lysosome compartment (LELC) positive for Lamp-2 (Fig 2A–2G). Borderline average values of 2xICQ are detected for ApoD colocalization with Caveolin-1 (Fig 2G), though some cells in the sample studied show clear colocalization over the threshold (Fig 2B). A significant PQ-dependent enrichment is observed in ApoD colocalization with Clathrin, Lamp-2 and LC3 at 24h of treatment (asterisks in Fig 2G and S2A–S2E Fig). At shorter times (2h) no PQ-dependent enrichment of ApoD is observed in the lysosomal or autophagosomal compartments of 1321N1 cells, while a prominent colocalization is seen for ApoD-Clathrin (asterisk in Fig 2H).

In opposition, ApoD is not detected in mitochondria or peroxisomes, two organelles involved in oxidative stress generation and management (Fig 2G and S2F–S2G Fig).

As expected for a Lipocalin, 1321N1 cells secrete ApoD to the culture medium (S3A Fig). Also, in ApoD-transfected human embryonic kidney-293T (HEK293T) cells, extracellular ApoD is detected with a stable accumulation in the culture medium over time (S3B Fig). However, no significant colocalization was observed with rough endoplasmic reticulum (RER; S3C Fig) and only borderline values were obtained with the Golgi apparatus (Fig 1A and 1G) of 1321N1 cells. A rapid passage through RER and Golgi in astroglial cells might render ApoD concentration below detection levels in those organelles, a hypothesis supported by the evident ApoD-RER colocalization obtained when ApoD is overexpressed in HEK293T cells (S3D Fig).

To further analyze the spatial domain of ApoD overlap with organelle markers we calculated a percent pixel overlap (referenced to the ApoD signal) as a parameter independent of the fluorescence intensity taken into account in ICQ (Fig 2I). The representation of Clathrin pits, early endosomes, LELC and autophagosomes within the ApoD spatial domain is quite high (18–30%), indicating that these organelles are common residence sites for ApoD. No enrichment in spatial overlap is detected upon PQ treatment, suggesting that the elevated ApoD 2xICQ values reported above (Fig 2G and 2H) represent a stress- and time-dependent increase in ApoD concentration in those stable spatial domains.

### ApoD is transiently enriched in the LELC upon oxidative stress, and subsequently transported to either autophagosomes or plasma membrane

**Fig 3:**
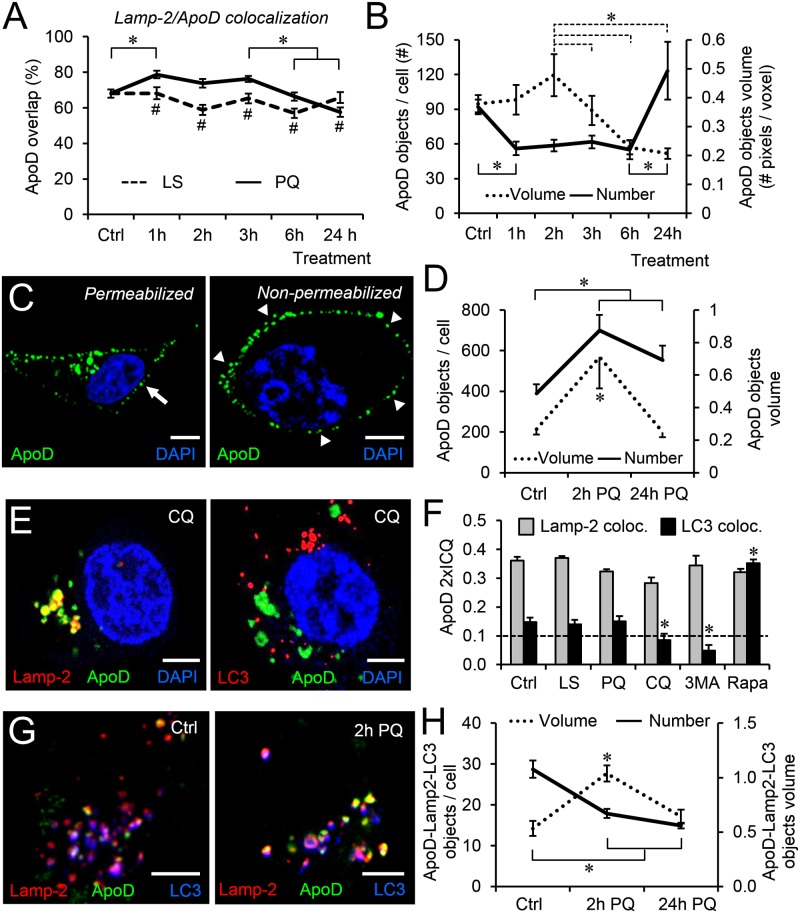
Lysosomal location of ApoD is followed by either entrance in autophagolysosomes or plasma membrane targeting. A. Spatial colocalization of ApoD with Lamp-2 (Overlap referenced to ApoD signal) in 1321N1 cells in control and PQ treatment over time. B. Number and volume of ApoD-positive objects along PQ treatment. C. Representative confocal sections of ApoD signal in permeabilized and non-permeabilized 1321N1 cells at 24h of PQ treatment. D. The number and volume of ApoD-positive membrane objects significantly differ with time of PQ treatment (Number: p = 0.002; Volume: p = 0.004). E. Representative confocal sections of 1321N1 cells labeled with ApoD and either Lamp-2 or LC3 after 1h of chloroquine (CQ) treatment, demonstrating that ApoD entrance in autophagolysosomes is dependent on lysosome-phagosome fusion. F. Colocalization index referenced to ApoD signal for LELC and autophagolysosomes in control and experimental conditions: 2h low serum (LS); 2h PQ; 1h CQ; 1h 3-methyladenine (3-MA) and 1h Rapamycin (Rapa). G. Representative confocal sections showing triple colocalization of ApoD, Lamp-2 and LC3 in control and 2h PQ treatment. H. Number and volume of ApoD/Lamp-2/LC3-positive objects in control and after 2 or 24h PQ treatment. Error bars in all graphs represent SEM (n = 20 cells/marker from at least two independent experiments). Object volume was measured by number of pixels/voxel in B, D and H. Statistical differences were assessed by ANOVA on Ranks (p<0.001) with Tukey post-hoc method (p<0.05, denoted by asterisks within variables, and by number sing between variables). Calibration bars in C,E,G: 5 μm.

Given the prominent colocalization of ApoD with the LELC marker Lamp-2 in 1321N1 cells, we performed a more detailed time-course analysis of their spatial overlap. PQ triggers specifically a significant and transient increase in pixel overlap (Fig 3A), which is accompanied by a change in the distribution of the ApoD signal (Fig 3B): an initial phase of large and less numerous ApoD objects is followed by a late phase with more objects of small average size. This analysis suggests a PQ-dependent early enrichment of ApoD in LELC that might coincide with organelle fusion, probably autophagolysosomes, followed by ApoD traffic to smaller vesicles.

Our colocalization studies, performed in conditions of membrane permeabilization (see Methods), uncovered that ApoD signal was also present in a peripheral dotted pattern after 24h of PQ treatment (Fig 3C, arrow in left panel). By performing ApoD immunolocalization in non-permeabilized conditions, we confirmed that this labeling is due to ApoD presence at the extracellular side of the plasma membrane (Fig 3C, arrowheads in right panel). Our object-based analysis of the plasma membrane located ApoD signal at 2 and 24h of PQ treatment (Fig 3D) shows two phases, which might be indicative of different cell biological processes: (i) An initial phase with increased number and size of objects is concordant with the early enrichment of ApoD in Clathrin-positive organelles (Fig 2H). (ii) Small, but still abundant, membrane-associated ApoD aggregates at 24h might point to a late traffic back to the membrane from the LELC.

The early distribution of ApoD signal in permeabilized cells upon PQ treatment (Fig 2B) suggests organelle fusion, and ApoD is a common resident in LC3-positive organelles of 1321N1 cells (Fig 2F). Therefore, we studied ApoD traffic in the autophagy process. As predicted by the Drosophila *in vivo* results, we show that ApoD entry in autophagolysosomes is dependent on a proper lysosome-autophagosome fusion since chloroquine (CQ), known to alkalinize lysosomal pH and prevent its fusion [51], completely abolished colocalization of ApoD with LC3 (Fig 3E). We then quantified the colocalization of ApoD with Lamp-2 or LC3 (2xICQ index referenced to ApoD signal; Fig 3F). The presence of ApoD in LELC is relatively stable, while colocalization with LC3 drops below random levels both, when lysosome-phagosome fusion is impaired by CQ or when autophagy initiation is blocked at an early step using 3-methyladenine (3-MA). Likewise, a significant increase in ApoD-LC3 colocalization is observed when autophagy is stimulated by the mTOR inhibitor Rapamycin (Rap).

An object-based analysis of ApoD-Lamp2-LC3 triple colocalization in 1321N1 cells exposed to PQ (Fig 3G and 3H), shows a transient increase in the volume of objects labeled by the three markers (ApoD-positive autophagolysosomes) and a decrease in their number. This suggests that a basal level of autophagic activity exists in these cells, and that after an initial phase of fusions of autophagolysosomes upon PQ treatment, autophagy is resolved with a net decrease of objects with the three markers. These data, together with the increased ApoD-LC3 colocalization after 24h PQ (Fig 2G) and a net PQ-dependent decrease in the number of LC3-positive vesicles (S4 Fig) suggest that ApoD is enriched in autophagolysosomes upon oxidative challenge.

**Fig 4:**
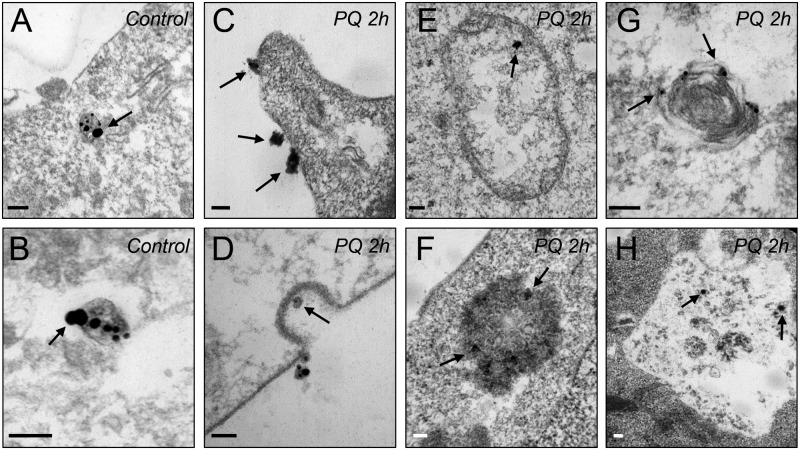
Subcellular localization of ApoD in 1321N1 cells in control and PQ conditions by immunoelectron microscopy. ApoD labeling is shown by means of silver-enhanced gold particles, some of them denoted by arrows. A. Localization of ApoD in early endosomes, near the plasma membrane, in control conditions. B. In control cells, ApoD locates at the membrane of small perinuclear lysosomes. C. ApoD is frequently found associated with the plasma membrane in PQ treated cells. D. Upon oxidative stress, ApoD is internalized through Clathrin-coated pits, identified by the characteristic inner plasma membrane coating at this particular location. E. In treated cells, ApoD is also found in large late endosomes. F. Under PQ conditions, ApoD is recruited to large and electron-dense late endosomal-lysosomal compartment. G. ApoD is also found in larger and mature secondary lysosomes, which contain particles undergoing digestion in the treated cells. H. ApoD was also located in autophagolysosomes, identified by their double membrane and a heterogeneous content with partially digested parts of cellular organelles. Calibration bars: 100 nm.

The subcellular localization of ApoD in 1321N1 cells in control and PQ conditions was further confirmed by morphological criteria of immunoelectron microscopy (Fig 4). In control conditions ApoD was mostly detected in early endosomes, small and located close to the plasma membrane (Fig 4A), as well as in lysosomes, electron- dense vesicles close to the nucleus (Fig 4B). Clear differences are observed upon PQ treatment in the representation of labeled subcellular localizations. After 2h of PQ treatment ApoD signal was observed on the plasma membrane (Fig 4C), being endocytosed in Clathrin-coated pits (Fig 4D), in larger late endosomes (Fig 4E), electron-dense lysosomes (Fig 4F), secondary lysosomes (Fig 4G), and autophagolysosomes (Fig 4H). In most cases, ApoD signal is observed associated to membranes. Interestingly, lysosomes under oxidative stress show an enrichment of membrane-associated ApoD (Fig 4F) compared to control conditions (Fig 4B).

### The late endosome-lysosome compartment is a “functional niche” for ApoD

**Fig 5:**
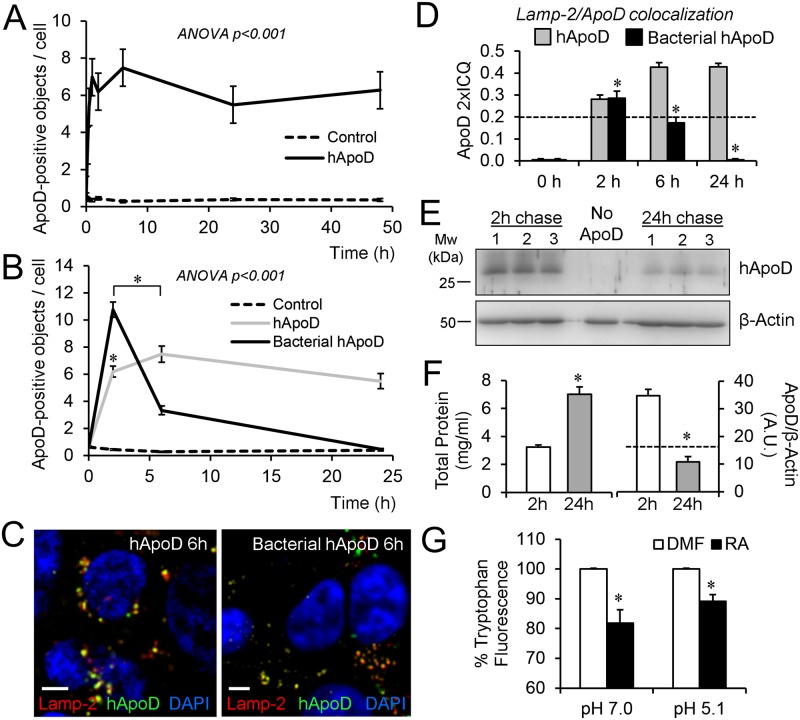
ApoD stability in LELC is glycosylation-dependent and its Lipocalin fold maintains lipid binding functionality in acidic pH. A. Number of ApoD-positive objects in HEK293T cells after exposure to 500 nM native human ApoD protein (2h pulse/48h chase). B. A 2h pulse/24h chase experiment is performed with bacterially-expressed ApoD protein. Control in A and B are HEK293T cells not exposed to ApoD. C. Colocalization of ApoD with Lamp-2 (6h chase) after exposure to either native human ApoD or bacterially-expressed human ApoD for 2h. D. Lamp-2 colocalization index referenced to ApoD signal (ApoD 2xICQ) in HEK293T in a 2h pulse/24h chase experiment with native or bacterially-expressed ApoD. E. Immunoblot analysis of ApoD loss rate in HEK293T subject to 2h pulse/24h chase with native human ApoD. Equal amounts of total protein (40 μg) are loaded from three independent cultures. A control culture with no ApoD addition is shown in the center lane. F. Total protein concentration duplicates during the experiment due to cell division (left graph). The dashed line marks (right graph) the concentration value expected for ApoD following dilution by cell division. G. Tryptophan fluorescence quenching analysis of retinoic acid (RA) binding to native human ApoD at neutral and acidic pH (pH value selected from peak of lysosomal-pH distribution upon 2h PQ treatment; see Fig 4D). Dimethylformamide (DMF) was used as carrier. Error bars represent SEM in all figures. A-D: n = 20 cells from at least two independent experiments. E-G: n = 3–5 independent experiments analyzed; asterisks represent significant differences (p<0.01) assessed by ANOVA with Holm-Sidak post-hoc method. Calibration bars in C: 5 μm.

Since the LELC and autophagolysosomal compartments participate in protein degradation, we tested whether the ApoD passage through them simply reflects its degradation pathway. We used cells not expressing ApoD (HEK293T) subjected to a 2h pulse-chase experiments with purified human ApoD [39]. After a 2h period of ApoD exposure, ApoD labeling was evaluated in cells for up to 48h. The intracellular content of ApoD was very stable along the experimental period when we used native human ApoD (Fig 5A). However, a bacterially produced non-glycosylated human ApoD is rapidly endocytosed and quickly degraded (Fig 5B). Colocalization with Lamp-2 (Fig 5C and 5D) shows that bacterial recombinant ApoD reaches the LELC, but the signal disappears with a fast time course. Using immunoblot, we have estimated that only 30% of endocytosed native human ApoD is lost during a division cycle in HEK293T cells (Fig 5E and 5F), and a fraction of that loss corresponds to ApoD secretion to the culture medium (S3B Fig).

These experiments demonstrate a very stable presence of native ApoD in the intracellular compartments analyzed above, and particularly in LELC, which suggests a functional role there. It is well known that proteins with essential functions within lysosomes bear carbohydrate shields against proteolysis [52]. In addition to the stable Lipocalin folding, ApoD N-linked glycosylation [53] might be responsible for such biochemical stability in protease-rich environments.

To further assay whether the Lipocalin folding can be stable in the acidic lysosomal lumen, we performed ligand binding assays at pH 7.0 and 5.1 (Fig 5G; pH chosen in light of the pH distribution of ApoD-positive lysosomes under stress conditions, see below). Binding to retinoic acid, a generic hydrophobic ligand known to bind all human Lipocalins tested so far [19], indicates that ApoD ligand binding is functional under acidic conditions.

### ApoD identifies two pH domains in the astroglial lysosomal compartment with differential responses to oxidative stress

An acidic pH is a defining functional property of lysosomes that allow their protease and lipase activities to be tightly controlled within the cell in addition to influence lysosomal fusions and traffic. We set to study the functional relationships of ApoD to lysosomal pH by using the membrane permeable LysoSensor Yellow/Blue DND-160, a ratiometric dye specifically targeted to all lysosomes, and not only those reaching the LELC through the endocytic pathway ([54,55]; see Methods and S5 Fig). We used either excitation analysis in cell populations (S5A Fig), suitable for pH 4.0–6.0, or emission spectral analysis of single lysosomes in confocal microscopy optical sections combined with ApoD immunodetection (S5B–S5D Fig; Linear fit for pH 4.0–5.5).

**Fig 6:**
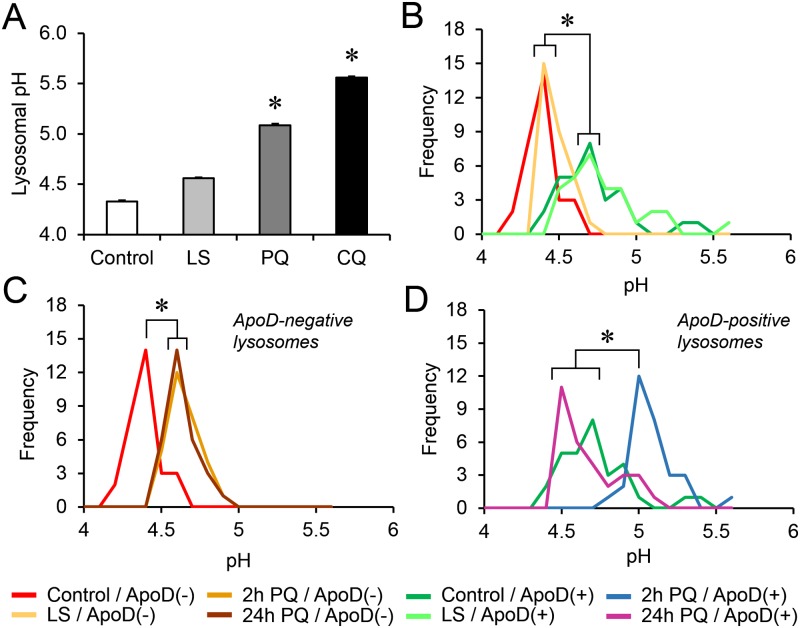
Single-lysosome pH measurements and ApoD labeling identify lysosomal populations with differential responses to oxidative stress. A. LysoSensor ratiometric excitation analysis of lysosomal pH in 1321N1 cell populations under LS (2h), PQ (2h), or CQ (1h) conditions (n = 30 cells/condition). Oxidative stress generated by PQ alkalinizes the lysosomal compartment. CQ was used as positive control for alkalinization. B-D. Frequency distribution of lysosomal pH measured by confocal emission spectra analysis in single lysosomes of 1321N1 cells. Immunolocalization of ApoD allows for the detection of ApoD-positive and negative lysosomes (n = 30 lysosomes/category). ApoD-positive and negative lysosomes in control and LS conditions are compared in B. ApoD-positive lysosomes distribution peak at pH 4.7 in both conditions, while that of ApoD-negative lysosomes is 4.4. The response of ApoD-negative lysosomes to PQ is shown in C. A small alkalinization (0.2 pH units) is established by 2h and maintained after 24h of PQ treatment. The response of ApoD-positive lysosomes to PQ is shown in D. ApoD is present in a subset of lysosomes specifically sensitive to oxidative stress that undergo a large (0.5 pH units) but reversible alkalinization in response to PQ. Differences were assessed by ANOVA on Ranks with Tukey post-hoc method (p<0.05, denoted by asterisks).

We first analyzed the effects of the treatments used in our experimental paradigm on the average lysosomal pH in 1321N1 cell populations (Fig 6A). Chloroquine (CQ) was used as positive control. Two hours of PQ treatment resulted in significant lysosomal alkalinization (with an average increase of 0.5 pH units), in agreement with the reported sensitivity of lysosomal membranes to oxidative stress [14] resulting in proton leakage that counteracts pH gradient generation mechanisms.

When pH was measured at the single organelle level combined with ApoD labeling, we discover a striking difference in the frequency distributions of pH values (Fig 6B). Lysosomes without ApoD show a narrow pH distribution with a frequency maximum at pH 4.4, while ApoD-positive lysosomes show a broaden distribution (range: 4.4–5.5) and a mode at pH 4.7 still in the range of lysosomal pH. This difference observed in control conditions is maintained when lowering serum in the culture medium. When we apply PQ, ApoD-negative lysosomes suffer a mild alkalinization (average peak shifts 0.2 pH units; Fig 6C) that is persistent after 24h of treatment. However, when the ApoD-positive lysosome pool is analyzed (Fig 6D), a larger pH increase is observed (average of 0.5 pH units). This alkalinization is transitory, since lysosomal pH distribution returns after 24h of PQ treatment to more acidic values, within the range of ApoD-positive lysosomes in control conditions. These data support the existence of subsets of lysosomes differing not only in their pH and ApoD content, but also in their sensitivity and response to an oxidative insult.

Our results show that ApoD is present in a subset of lysosomes specifically sensitive to oxidative stress that undergo a large but reversible alkalinization in response to PQ. Is ApoD responsible for these changes or it locates in lysosomes passively subjected to the PQ-triggered pH shifts?

### ApoD has an active role in the oxidation-sensitive lysosomal subdomain of cells, including neurons and astrocytes

**Fig 7:**
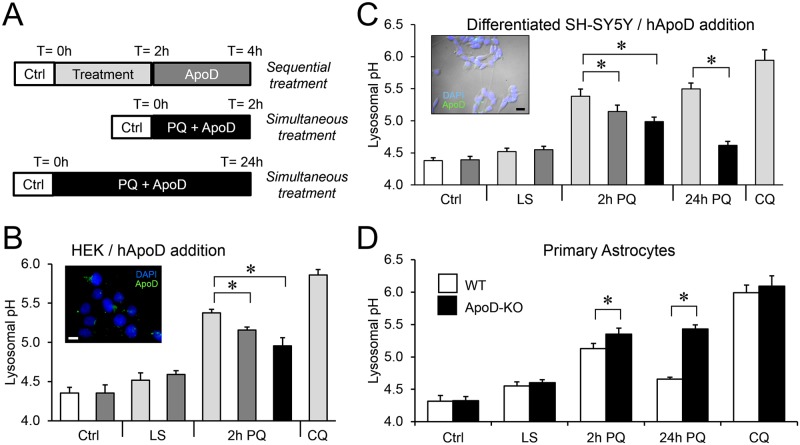
ApoD prevents and reverts oxidation-dependent lysosomal alkalinization in cell lines, differentiated neurons and primary astrocytes. A. Graphic representation of cell treatment protocols used to analyze lysosomal pH changes. B. HEK293T cells (not expressing ApoD) are treated with exogenous ApoD and PQ (short simultaneous or sequential applications). ApoD is able to prevent (black bar) and to revert (dark grey bars) the PQ-induced lysosomal alkalinization. C. In differentiated SH-SY5Y neurons (also without ApoD expression), we used short (2h) interval protocols, as in HEK293T cells, and a long (24h) simultaneous ApoD-PQ treatment. ApoD is able to modify neuronal lysosomal pH (dark grey bars) and to re-acidify oxidized lysosomes (black bars). This protective effect is durable over the time period studied, and reversion is complete by 24 h. D. Primary WT and ApoD-KO astrocytes (without exogenous ApoD addition) show PQ-dependent lysosome alkalinization, but only WT astrocytes are able to return to the basal pH level. The control CQ treatment in B-D results in lysosomal pH values at the edge of the LysoSensor probe dynamic range. Insets in B and C show representative immunocytochemistry images of exogenous ApoD endocytosed by ApoD-non-expressing cells. Differences in B-D were assessed by ANOVA on Ranks with Tukey post-hoc method (p<0.05, denoted by asterisks). Calibration bars in B,C: 20 μm.

To test whether there is a causal relationship between ApoD presence and lysosomal pH we added exogenous purified human ApoD to non-expressing HEK293T cells, either simultaneous to PQ exposure (2h treatment) or sequentially, by first provoking lysosomal alkalinization with PQ (2h) and then adding exogenous ApoD (Fig 7A). We have already shown that by 2h ApoD has entered the LELC in HEK293T cells (Fig 5D). The addition of ApoD prevents the PQ-triggered alkalinization and is able to reverse an already established effect of PQ (asterisks Fig 7B).

**Fig 8:**
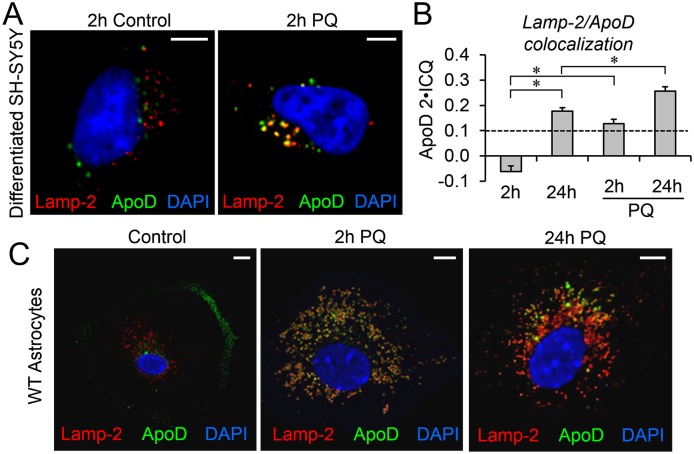
ApoD is targeted to lysosomes in an oxidative stress-dependent manner in differentiated neurons and primary astrocytes. A. Representative fluorescence microscopy images of ApoD and Lamp-2 in differentiated SH-SY5Y neurons after 2h exposure to hApoD with or without simultaneous PQ treatment. B. Average colocalization index referenced to ApoD signal (ApoD 2xICQ) in neurons treated with hApoD in control and PQ conditions. Significant colocalization is observed in control conditions only 24h post-ApoD exposure. Rapid entry into lysosomes is observed upon PQ treatment at 2h. The dotted line represents the colocalization threshold. Statistical differences (asterisks) were assessed by two-way ANOVA, Holm-Sidak post-hoc method (p<0.01). C. Representative fluorescence microscopy images of ApoD and Lamp-2 in primary WT murine astrocytes in control and PQ conditions. ApoD distribution changes dramatically from membrane labeling (concentrated in lamellae) to intracellular organelles showing a time-dependent LELC colocalization. Calibration bars in A,C: 5 μm.

We also added purified ApoD to differentiated SH-SY5Y neurons (Fig 7C). Here a longer simultaneous ApoD-PQ treatment was applied due to the dynamics of ApoD entry into neuronal LELC (see below, Fig 8). Again, ApoD is able to prevent (particularly prominently in the long 24h treatment), as well as to revert, the alkalinizing effects of PQ (asterisks in Fig 7C). Lack of ApoD labelling in the absence of exogenous addition of hApoD indicates that SH-SY5Y neurons do not express ApoD, as has been recently confirmed by RNAseq techniques (http://systemsbiology.uni.lu/shsy5y/). Therefore, the observed rescue of lysosomal pH can only be attributed to an endocytosis-mediated mechanism (Fig 8).

Aside of modifying ApoD presence by exogenous addition, we cultured murine primary astrocytes from wild type (WT) or knock-out (ApoD-KO) mice (Fig 7D). Using this model, we demonstrate: 1) The PQ-dependent alkalinization and re-acidification of 1321N1 lysosomes is also present in WT primary astrocytes. 2) ApoD-KO lysosomes do increase their pH upon oxidative stress, therefore indicating that the alkalinization itself is not related to ApoD presence. 3) No re-acidification is achieved in the absence of ApoD.

Summarizing, our data show that ApoD is present in a particular subset of PQ-sensitive lysosomes, which inevitably alkalinize in the presence of oxidative stress, an effect known to be due to lysosomal membrane damage [14,56]. We also show that ApoD is responsible for the pH recovery of PQ-challenged lysosomes. These data support the hypothesis that lysosomal membrane recovery is compromised in the absence of ApoD, and that this Lipocalin, with its structure and lipid-binding properties preserved, contributes to the repair of damaged lysosomal membranes in astrocytes and neurons.

### ApoD is targeted to the lysosomal compartment in an oxidative stress-dependent manner, both in differentiated neurons and primary astrocytes

We have shown that ApoD affects the differential response of a subset of lysosomes to oxidative stress by actively promoting pH recovery. Then, is the entrance of ApoD to the lysosomal compartment a constitutive or a regulated process?

A Lamp-2/ApoD colocalization analysis in differentiated SH-SY5Y neurons after a two-hour pulse of exogenous human ApoD (Fig 8A and 8B) shows that, in contrast with the observed rapid entrance into the LELC compartment in HEK293T cells (Fig 5), differentiated neurons show no colocalization in control conditions 2h after ApoD addition (Fig 8A and 8B). Colocalization is evident later on, after a 24h chase (Fig 8B). In contrast, a significant colocalization is observed at 2h in the presence of PQ, and keeps increasing during the 24h post-ApoD supplementation period (Fig 8B). Thus, ApoD entry into lysosomes in neurons is dependent on stress conditions, being slow in the absence of stress but very quick in its presence.

A similar phenomenon is observed with native murine ApoD in primary astrocytes (Fig 8C), though with a remarkable feature: In control condition WT astrocytes show a clear predominant plasma membrane localization of ApoD and no colocalization with Lamp-2. ApoD entrance into the LELC is PQ-dependent and has a fast time course, with a substantial colocalization at 2h.

**Fig 9:**
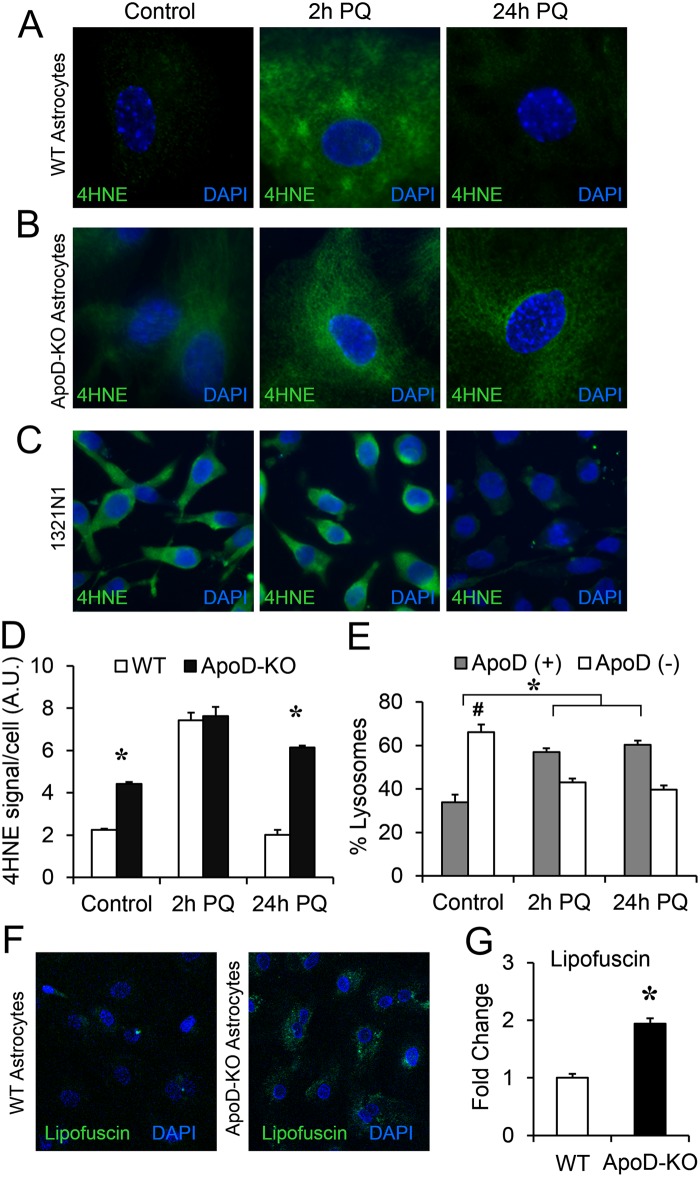
ApoD expression and targeting to lysosomes prevent cell lipid peroxidation and lysosomal accumulation of oxidized cargo. A-B. Immunodetection of 4HNE adducts by fluorescence microscopy in primary WT (A) or ApoD-KO (B) astrocytes in control and PQ conditions. D. Representative fluorescence microscopy images of 4HNE labeling in 1321N1 cells in control and PQ conditions. Basal oxidative stress level is high compared to primary astrocytes. Long-term response to PQ results in clearance of 4HNE adducts at 24h. D. Plot representing 4HNE raw intensity/cell (n>400 cells/genotype/condition) in primary astrocytes. WT astrocytes have very low basal 4HNE levels and recover completely after 24h of PQ treatment. Without ApoD, basal lipid peroxidation is significantly high and astrocytes are unable to counteract PQ long-term effects. E. Lysosome quantification using LysoSensor combined with ApoD immunolabeling in 1321N1 cells. The proportion of ApoD-positive and negative lysosomes are shown (n = 30 cells/condition). PQ triggers a significant enrichment in ApoD-positive lysosomes and depletion of ApoD-negative lysosomes. F. Representative fluorescence microscopy images of lipofuscin signal in WT and ApoD-KO astrocytes under control conditions. G. Plot representing the fold change in lipofuscin signal measured by confocal spectral analysis in ApoD-KO astrocytes compared to WT cells (n>1500 astrocytes/genotype). Statistical differences in C and E were assessed by two-way ANOVA (p<0.001), and Holm-Sidak post-hoc method (p<0.001). Differences in G were assessed by Student’s t-Test. Calibration bars: 20 μm.

We can conclude that the accelerated targeting of ApoD to the lysosomal compartment of neurons and primary astrocytes under stress conditions is a regulated process. To further explore the consequences of this process we measured the levels of 4-hydroxynonenal (4HNE) (Fig 9A, 9B and 9D), a lipid peroxidation-derived adduct, as a proxy for oxidative stress in each experimental condition. Interestingly, primary WT astrocytes have very low 4HNE levels in control conditions; they increase their oxidation levels by 2h of PQ treatment, but return to basal levels by 24h (Fig 9A and 9D). By contrast, ApoD-KO astrocytes show a significant basal level of oxidative stress and fail to recover from the insult (Fig 9B and 9D), further demonstrating the reported protective role of ApoD upon oxidative stress [26]. Starting with a higher oxidation state, the level achieved by 2h PQ in ApoD-KO astrocytes matches WT levels, indicating that cells have reached a 4HNE maximum. Therefore, ApoD entrance into lysosomes of WT astrocytes occurs at the peak of oxidative stress.

If ApoD targeting to lysosomes is regulated by oxidative stress, we wondered why there is a significant colocalization of ApoD with Lamp2 in 1321N1 astrocytoma cells under control conditions (Figs 2–4). Fig 9C demonstrates that 1321N1 cells have a high basal oxidative stress, coherent with the high metabolic rate of cancer cells. Thus, ApoD is targeted to lysosomes under these conditions. The levels of 4HNE further increase in response to PQ treatment, and are followed by a substantial clearance of 4HNE adducts. This rebound effect is consistent with the pH recovery effect observed in our single-lysosome study (Fig 6D), and suggests that oxidative-challenged cells transiently activate protective mechanisms (ApoD among them) that clear lipid peroxidation products. An additional analysis of the 1321N1 single lysosome data (Fig 9E) shows a low proportion of ApoD-positive lysosomes in control conditions, and a significant early increase upon PQ treatment that is maintained by 24h (overriding the proportion of ApoD-negative lysosomes). Thus, the mechanism controlling lysosomal targeting of ApoD also occurs in the astrocytoma cell line 1321N1. The efficient clearance of oxidized products might be the result of a very efficient lysosomal function, contributed by the ApoD-dependent re-acidification after PQ insult.

An active role of ApoD in oxidized lysosomes was further demonstrated by measuring lipofuscin, a reported readout of lysosomal pH dysfunction that results in cellular accumulation of damaged oxidized macromolecules [57]. A spectral analysis of confocal microscopy images of WT and ApoD-KO primary astrocytes (Fig 9F and 9G) shows that the absence of ApoD generates a significant increase in lipofuscin signal in astrocytes.

### ApoD expression and targeting to lysosomes prevent lysosomal permeabilization

**Fig 10:**
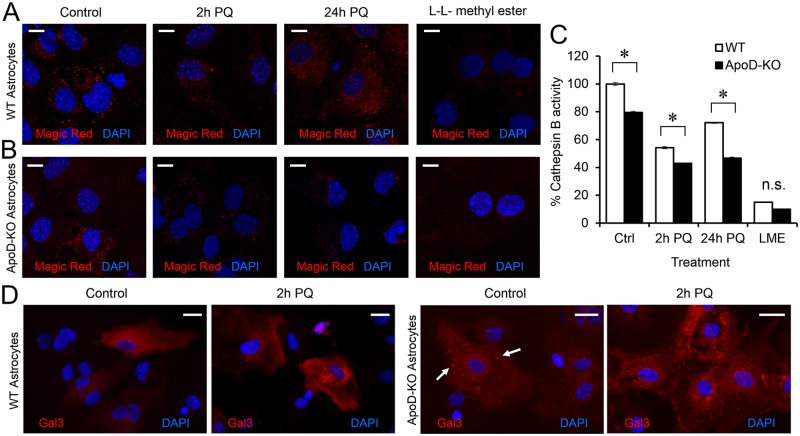
ApoD prevents lysosomal membrane permeabilization upon oxidative stress. A-B. Cathepsin B activity monitored by Magic Red assay. The punctate fluorescent signal is proportional to its proteolytic activity, taking place in lysosomes. WT (A) and ApoD-KO (B) primary astrocytes are compared. L-leucyl-L-leucine methyl ester (LLME) is used as positive control for lysosomal membrane rupture. C. Cathepsin B activity is plotted normalized to values obtained in control WT astrocytes. PQ provokes a reduction of Cathepsin B activity that is recovered after 24 h treatment in WT astrocytes. Lack of ApoD results in reduced basal activity and unrecoverable activity loss after PQ insult. D. Representative fluorescence microscopy images of Galectin-3 signal in WT and ApoD-KO astrocytes under control and 2 h of PQ treatment. A switch from cytoplasmic to vesicular (lysosomal) Galectin-3 labeling occurs under oxidative stress conditions in WT astrocytes. The lysosomal labeling of Galectin-3 is evident for ApoD-KO primary astrocytes in control conditions (arrows), and increases under PQ treatment. Statistical differences in C were assessed by two-way ANOVA (p<0.001), and Holm-Sidak post-hoc method (p<0.001). Calibration bars: 20 μm (A), 10 μm (B).

If the mechanism by which ApoD controls lysosomal pH is due to lysosomal membrane stabilization, two predictions can be made for lysosomal behavior in the absence of ApoD: 1) lysosomal proteases would decrease their activity, and 2) cytoplasmic proteins would aberrantly enter lysosomes. A Cathepsin B activity assay (Fig 10A–10C) demonstrate the first prediction. In WT astrocytes (Fig 10A and 10C), PQ-triggered oxidation reduces Cathepsin B activity, but a clear recovery takes place upon prolonged PQ exposure in parallel with lipid-peroxide clearance (Fig 9A and 9C). In the absence of ApoD (Fig 10B and 10C), Cathepsin B activity is significantly reduced in basal conditions, further deteriorated upon PQ treatment, and no recovery is obtained after 24 h treatment.

Galectin-3 immunocytochemistry (Fig 10C) demonstrate the second prediction. This lectin shows a diffuse cytoplasmic location in control WT astrocytes cultures, but translocates to leaky lysosomes undergoing oxidative stress-dependent membrane permeabilization upon PQ insult, giving a punctate labeling pattern [58]. Astrocytes lacking ApoD show evident Galectin-3 puncta in control conditions (arrows in Fig 9H), and the effect is further increased upon 2h PQ.

In summary, entrance of ApoD into the LELC, and particularly into lysosomes, is actively promoted in pro-oxidative conditions in neurons and astrocytes. Our data demonstrate that ApoD, contributing to lysosomal pH recovery upon oxidative stress, is recruited to a vulnerable subset of lysosomes, where it helps to keep lipid peroxides levels under control and to safeguard lysosomal functional integrity by avoiding lysosomal membrane permeabilization.

These findings represent a novel function for ApoD, and for Lipocalins. Their active role in the lysosomal compartment provides a clear explanation for the mechanism of GLaz rescue of polyglutamine-based neurodegeneration [37]: it requires fusion of autophagosomes to healthy lysosomes to optimize autophagy (Fig 1). Moreover, we have reported an protective role for ApoD in the functional recovery of injured mammalian peripheral nerves by a mechanism regulating myelin phagocytosis efficiency [32]. Taking into account our current results, the mechanistic link between these two apparently unrelated biological processes is clear: The direct control by ApoD of lysosomal function efficiency.

We have centered this work on astrocytes, the front line of defense against oxidative stress and one of the nervous system cell types that express ApoD. Since ApoD conditions the pH-dependent functionality of the lysosomal compartment, how does it affect biological processes where a lysosomal optimal function is important for astrocytes?

### The lysosomal function of ApoD modifies the dynamics of myelin phagocytosis by astrocytes

Recent studies reveal that astrocytes have phagocytic functions [59,60,61]. They digest the phagocytosed cargo through a process regulated by lysosome pH levels and autophagosome-lysosome fusion [62]. This is a slow process in astrocytes, and is proposed to regulate antigen presentation by these cells through lysosomal fusion to the plasma membrane. We have shown that lysosomal ApoD either enters autophagolysosomes or traffics back to the plasma membrane (Fig 3), and that lysosomal pH depends on the presence of ApoD (Figs 6 and 7). Therefore, ApoD might be a candidate regulator for the “digest-or-present” process in astrocytes.

**Fig 11:**
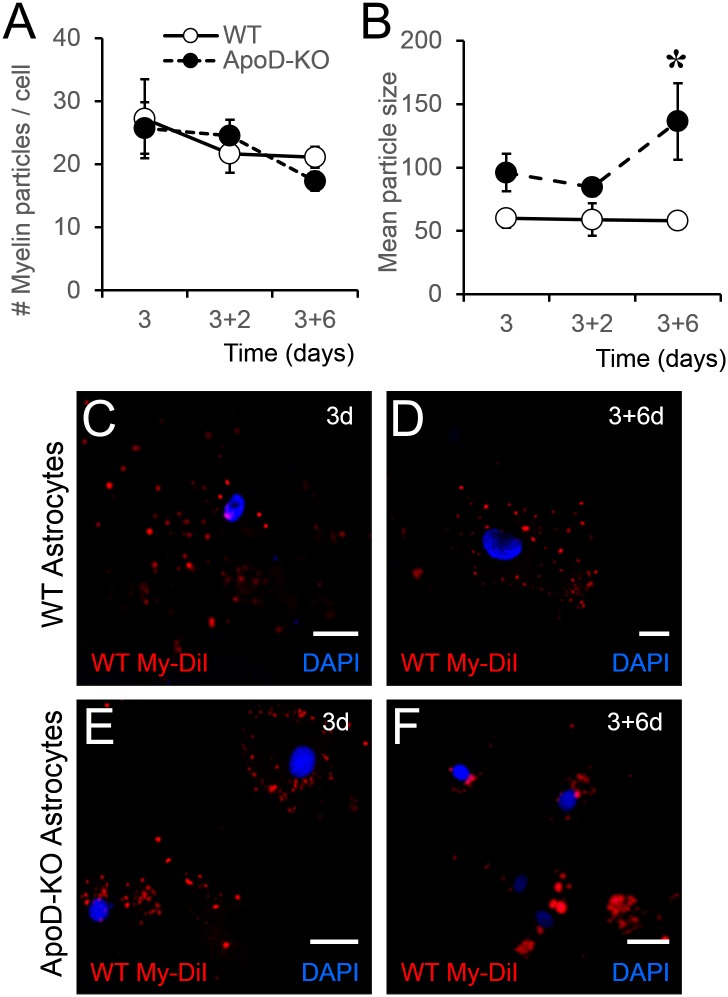
ApoD presence in mouse astrocytes is required for adequate processing of phagocytosed myelin. WT and ApoD-KO astrocytes were exposed to DiI labeled myelin for 3 days, and DiI signal was evaluated by fluorescence microscopy 2 and 6 days after removal of myelin. Three-way ANOVA was used to evaluate variable interactions, followed by two-way ANOVA to detect the origin of differences. A. Number of DiI-myelin particles phagocytosed by primary WT and ApoD-KO astrocytes. No differences are found between genotypes, indicating comparable initial levels of phagocytic activity. B. Mean particle size of DiI-positive objects is dependent on time of treatment (p = 0.032, three-way ANOVA). The values at 6 days post-myelin removal account for the difference (p<0.001, Holm-Sidak method). Only ApoD-KO astrocytes show a significant increase in large myelin particles. C-F. Representative images of DiI-myelin signal in primary WT or ApoD-KO astrocytes after myelin exposure (3d; C,E) and 6 days after myelin removal (3+6d; D,F). Calibration bars: 20 μm.

Astrocytes are reported to start degrading phagocytosed cargo in acidic lysosomes at 6h after exposure to cell debris [62]. We thus exposed primary astrocytes to DiI-labeled myelin for 3 days and monitored DiI signal at 2 and 6 days after myelin removal (Fig 11). We estimated the phagocytosis potential of WT and ApoD-KO astrocytes by measuring the number and size of DiI-labeled myelin particles (see Methods). Starting with comparable initial levels of phagocytic activity (Fig 11A; no differences are observed in the number of myelin particles phagocytosed during the 3h exposure period), both astrocyte genotypes decrease the number of particles over time. However, this reduction in numbers is accompanied by a significant increase in large myelin particles at 6 days post-myelin exposure in ApoD-KO astrocytes only (Fig 11B, 11E and 11F), indicating that phagocytosis resolution is impaired in the absence of ApoD. These results agree with the high load of phagocytosed material observed in alkaline astrocyte lysosomes [62]. Taking into account that myelin phagocytosis induces ROS production [63], the more alkaline ApoD-KO lysosomes are expected to be less efficient, resulting in delayed myelin degradation. No differences are found at earlier times, suggesting that the absence of ApoD results in a deregulated processing of the already ingested myelin, requiring the maintenance of lysosomal optimal pH and functionality.

## Discussion

**Fig 12:**
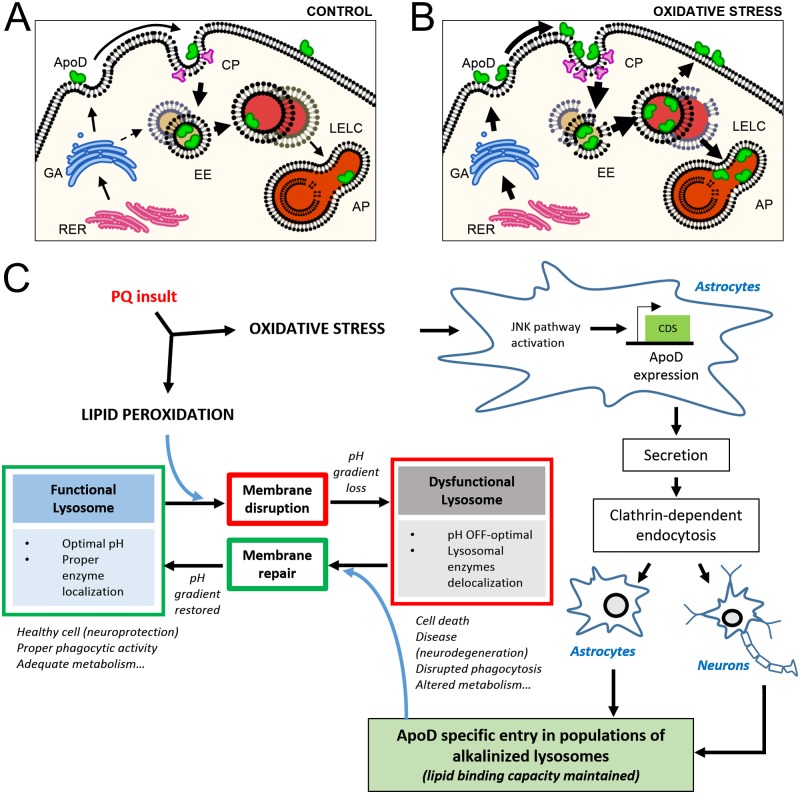
ApoD-dependent lysosomal integrity protection mechanism. A-B. Model of oxidative stress-dependent cellular traffic of ApoD. ApoD is depicted as a green molecule. Oxidized membranes are represented as broken lipid bilayers. ApoD enters only in a subset of lysosomes and restores their damaged membrane. C. PQ-triggered oxidative stress causes dysfunctions in a subset of vulnerable lysosomes, and, at the same time, induces expression, secretion, endocytosis and targeting of ApoD to that particular subset of lysosomes. ApoD is necessary and sufficient to restore lysosomal membrane and reinstate optimal lysosomal pH.

This study reveals for the first time a functionally complex stress-dependent traffic of the Lipocalin ApoD (Fig 12A and 12B) that is the base of a lysosomal protecting mechanism previously unknown (Fig 12C). While plasma membrane and endosomes are typical cellular locations for ApoD secreted under basal conditions (Fig 12A), lysosomes become an essential and stable niche for ApoD in a cell suffering from oxidative stress (Fig 12B). After a fast protein secretion through the canonical RER-Golgi pathway, stress conditions trigger ApoD endocytosis. Clathrin-dependent endocytosis is particularly favored early under oxidative stress conditions. ApoD then moves through the early endosomal compartment to reach the LELC. Subsequently, Lamp-2/ApoD-positive organelles either enter the autophagy pathway (early after oxidative stress stimulus), or ApoD is targeted back to the membrane at a late phase, possibly travelling within secretory lysosomes.

Our results explain important aspects of the ApoD neuroprotective mechanism previously unanticipated. 1) ApoD locates in a subset of lysosomes particularly sensitive to oxidative stress. These results support previous work reporting a functional heterogeneity of lysosomes according to their pH, vulnerability, pro-oxidative activity, or position within the cell [14,17,64]. 2) ApoD stability within lysosomes is dependent on its glycosylation state, which is functionally relevant given its reported heterogeneity in different tissues and cell types [65]. This result could also explain the absence of neuroprotective effects of bacterial recombinant ApoD against Aβ-challenged neuronal cells [66]. 3) ApoD targeting to lysosomes is a controlled process promoted by oxidative stress in ApoD-expressing astrocytes and ApoD-non-expressing neurons, which explains both autocrine and paracrine protective effects [26,36,37]. 4) ApoD behaves as an acute phase protein, finely tuned through a JNK pathway-dependent transcriptional expression [26,44] coordinated with a stress-dependent accelerated entry into lysosomes.

Another remarkable finding derived from our experiments is a functional link between the presence of intact ApoD within lysosomes and their pH. ApoD shows very low expression and seldom locates inside lysosomes of primary astrocytes cultured in control conditions. It is also found with more probability in endosomes than in lysosomes of control 1321N1 cells. However, it is rapidly up-regulated and mostly found in lysosomes upon oxidative stress. This situation is achieved in native cells after just 2h of PQ treatment, though it occurs basally in rapidly proliferating cell lines like the 1321N1 astroglioma cells. Oxidative stress makes ApoD to translocate quickly and specifically to lysosomes with a slightly alkalinized pH in basal conditions and high sensitivity to oxidation, which underlie their functional vulnerability. Nevertheless, the presence of ApoD in these lysosomes is necessary and sufficient for restoring lysosomal pH to normal values after oxidation-dependent alkalinization, as confirmed by the stably alkalinized PQ-challenged ApoD-KO cells. This result holds for cells that endogenously express ApoD, like astrocytes, and for non-expressing cells exogenously supplied with ApoD, like neurons.

Lysosomal alkalinization is known to result from ROS-induced membrane permeabilization [67], and we find clear signs of lysosomal dysfunction and membrane permeabilization in ApoD-KO lysosomes (Fig 10) together with high levels of lipid peroxidation that can be counteracted only in the presence of ApoD expression (Fig 9A–9D). Complex processes like bidirectional protein traffic along the endosome-lysosome compartments, or altered transcription, translation or trafficking of proton pumps to the lysosome, could result in pH changes similar to the observed ones. However, the time course of ApoD effects on lysosomal pH and the evidences of lysosomal permeabilization strongly support a direct effect of ApoD on lysosomal membranes. Such a mechanism is concordant with ApoD biochemical properties, its lipid binding properties (preserved at acid pH in the range of ApoD-positive lysosomes; Figs 5 and 6), and its membrane association, including lysosomal membranes (Fig 4). As a lipid peroxidation counteracting agent [25], ApoD restores the integrity of damaged lysosomal membranes (Fig 12C).

It is of special interest to compare the beneficial repair of lysosomal function by ApoD with the effects of other extracellular lipid binding proteins known to have effects on lysosomes. The Lipocalin Lcn2 reduces lysosomal degradative activity, resulting in insulin resistance in cardiomyoblasts [68]. Apolipoprotein E (also expressed by astrocytes and consistently related to neurodegenerative phenotypes) has deleterious effects on lysosomal function, as the ApoE4 allele causes lysosomal leakage and apoptosis [69].

That ApoD helps to maintain H^+^ gradients under oxidative stress in glial and neuronal lysosomes is a finding with high explanatory value in the understanding of lysosomal mechanisms of protection and of ApoD function. Lysosomal dysfunction does compromise cell resistance to oxidative stress, the major phenotypic hallmarks of all loss-of-function manipulations performed so far with ApoD and its related Lipocalins in animal models and cellular systems [26,29,33,36,38,39]. Moreover, a failure of lysosomal function is linked to inefficient toxic protein clearance in proteinopathies like SCA1 that ultimately leads to cell death and neurodegeneration. This study explains not only why neurodegeneration rescue by GLaz [37] depends on the lysosome-autophagosome fusion (Fig 1), but also why phagocytosis resolution in astrocytes (Fig 10) and after peripheral nervous system injury [32] is compromised. Interestingly, a recent report shows a similar delay in clearing myelin from injured nerves when lysosomal function is inhibited [70].

### Conclusions

This study demonstrates that ApoD contributes significantly to the evolutionarily conserved mechanism of protecting cells by protecting their lysosomes. ApoD could be the first lysosomal marker known to be specific for a particular subset of lysosomes: the most vulnerable to oxidative stress. Therefore, ApoD localization assessment should provide a useful tool for characterizing many physiological and pathological situations.

Our work focuses on the endogenous mechanisms of protection in the nervous system, where astrocytes are central players and neurons are especially vulnerable cells. However, the known functional pleiotropy of ApoD warrants that it will be relevant to many other biological processes and pathological situations. Understanding ApoD actions in lysosomes will open the possibility of manipulating this mechanism for therapeutic purposes, using ApoD as a carrier to reach the lysosome.

Although previously considered to play a lipid transport function in various body fluids, the Lipocalin ApoD can now be recognized as a relevant acute phase protein contributing to the nervous system response to stress, injury, neurodegeneration and aging by stabilizing the membrane of vulnerable lysosomes. This finding identifies a new lipid binding protein-dependent cellular mechanism by which lysosomes are functionally protected against oxidative stress.

## Materials and methods

### Cell culture and treatments

The cell lines 1321N1, HEK293T, and SH-SY5Y were obtained from Sigma-Aldrich and ATCC. Cells were grown at 37°C in humidity-saturated atmosphere containing 5% CO_2_. The culture medium was replaced twice a week, and cells were subcultured at 90% confluence.

The human astrocytoma cell line 1321N1 was cultured in Dulbecco-modified Eagle's medium (DMEM; Lonza), supplemented with heat-inactivated 5% fetal bovine serum (FBS), 1% L-glutamine (final concentration 2 nM), and 1% P-S-A stock (final concentration: 100 U/ml penicillin, 100 U/ml streptomycin, 0.25 μg/ml amphoterycin B).

The human neuroblastoma cell line SH-SY5Y was cultured in DMEM supplemented with 4.5 g/l glucose, heat-inactivated 10% FBS, 1% L-glutamine, 1% P-S stock (final concentration: 100 U/ml penicillin, 100 U/ml streptomycin) and 1% nonessential amino acids (Lonza). SH-SY5Y cells differentiation was achieved by culture on collagen-treated plates with medium supplemented with 3% FBS and Retinoic acid (10 μM). A 72h differentiation period was allowed before experiments.

HEK293T cells were cultured in DMEM supplemented with 4.5 g/l glucose, 1% L-glutamine, 1% P-S-A, and 10% FBS.

For the exogenous addition of ApoD, human ApoD purified from breast cystic fluid [39] or recombinant human ApoD from *E*. *coli* (ProSpec) were added (10 nM) to the cell cultures for 2h.

Cells treated with Paraquat (PQ; 500 μM; 1-24h), Chloroquine (CQ; 20 μM; 1h), Rapamycine (Rap; 2 μM; 2h), and 3-Methyladenine (3-MA; 5 mM; 2h) were cultured in phenol red-free DMEM supplemented with 1% L-glutamine, 1% P-S, and 0.2% charcoal stripped FBS. This medium without additives was used as our low-serum (LS) condition.

The expression constructs used in this work (pcDNA3.1-ApoD; pHSVer-GA; pHSVGA-cox8; pCDNA3.1-tgoGAm) were transiently transfected into cell lines using Lipofectamine LTX reagent (Invitrogen) according to the manufacturer’s protocol.

### Primary astrocyte cell cultures

ApoD-KO mice were generated by homologous recombination [29] maintained in positive pressure-ventilated racks at 25±1°C with 12 h light/dark cycle, fed *ad libitum* with standard rodent pellet diet (Global Diet 2014; Harlan Inc., Indianapolis, IN, USA), and allowed free access to filtered and UV-irradiated water. In order to avoid potential maternal effects of ApoD, and to generate WT and ApoD-KO mice of homogeneous genetic background, the experimental cohorts used in this study are the F1 generation of homozygous crosses of ApoD −/− and ApoD +/+ littermates born from heterozygous crosses of an ApoD-KO line backcrossed for over 20 generations into the C57BL/6J background.

We used neonatal (0–1 days old) mice of two genotypes: ApoD-KO and their WT littermates. Cerebral cortices were quickly extracted, their meninges removed by rolling on a sterile filter paper, and pieces of cortex placed in Earle’s Balanced Salt Solution (EBSS) containing 2.4 mg/ml DNAse I and 0.2 mg/ml bovine serum albumin (BSA). Tissue was minced with a surgical blade; centrifuged (200 g, 2 min); incubated with 10 mg/ml trypsin for 15 min at 37°C (incubation terminated by 10% FBS addition); mechanically dissociated with a Pasteur pipette and centrifuged (200 g, 5 min). The last two steps were repeated, and the resulting cells were resuspended in DMEM with 10% FBS, 1% L-glutamine, 1% P-S-A. Cells were plated onto culture flasks and incubated at 37°C in 5% CO_2_ with 90–95% humidity, and the culture medium was replaced weekly. Cell cultures were used for experiments after two subculture steps, when >99% of cells are astrocytes [26].

### Immunocytochemistry

Cells attached to poly-L-lysine (SIGMA) treated coverslips were fixed with 4% phosphate-buffered formaldehyde. Following washes in phosphate-buffered saline (PBS), the cells were blocked and permeabilized with Tween-20 (0.1%) and 1% non-immune (goat or donkey) serum. Cells were incubated overnight at 4°C with the following primary antibodies. Rabbit serum anti-human ApoD [custom made by Abyntek Biopharma against purified ApoD [39], or generated by Dr. C. López-Otin]. Goat serum anti-mouse ApoD (Santa Cruz Biotechnology). Mouse serum anti-clathrin LCA (Santa Cruz Biotechnology). Mouse serum anti-caveolin-1 (Santa Cruz Biotechnology). Goat serum anti-catalase (Santa Cruz Biotechnology). Mouse serum anti-EEA1 (BD Biosciences). Mouse serum anti-LC3 (MBL). Rat monoclonal anti-Galectin-3 (American Type Culture Collection, ATCC). Goat serum anti-4HNE (Alpha Diagnostic).

For immunolabeling with mouse monoclonal anti-human Lamp-2 and rat monoclonal anti-mouse Lamp-2 (DSHB), cells were blocked with 1% BSA, 10% normal goat serum, and 0.1% saponin in PBS, and incubated with primary and secondary antibodies for 1 h at room temperature.

Alexa Fluor 594 and 488 (Jackson Labs) or DyLight 405 (Thermo Scientific)-conjugated IgGs were used as secondary antibodies for fluorescence immunocytochemistry. After washes in PBS, the preparations were mounted with EverBrite Mounting Medium with DAPI, and sealed with CoverGrip Coverslip Sealant (Biotium).

### Immunoblot analysis

Cell lysates, or culture media (either directly or concentrated twenty times by 0.22 μm filter centrifugation), were collected to analyze the amount of ApoD. Immunoblot analyses were performed with proteins transferred to PVDF membranes using standard procedures, and exposed to rabbit serum anti-human ApoD and HRP-conjugated goat-anti-rabbit (Santa Cruz Biotechnology). An HRP-conjugated anti-β actin antibody (Sigma) was used to normalize protein loads. Membranes were developed with ECL reagents (Millipore), and the signal visualized with a digital camera (BioRad). The integrated optical density of the immunoreactive protein bands was measured in images taken within the linear range of the camera, avoiding signal saturation.

### Ligand binding assay by tryptophan fluorescence titration

These methods were performed as previously described [39]. Fluorescence measurements were conducted with a Shimadzu RF-5301PC spectrofluorometer in a quartz cuvette (105.251-QS, 3-mm path length; Hellma). Temperature was held at 22±0.1°C. Excitation wavelength was 295 nm (selective for tryptophan residues). Emission was recorded at 327–400 nm with slit width set at 5 nm. Purified human ApoD was diluted to 0.5 μM with 10 mM phosphate buffer (binding at pH 7.0), or 30 mM sodium citrate (binding at pH 5.1). The ligand retinoic acid (RA) was dissolved in dimethylformamide (DMF; Sigma). The mixture was equilibrated for 3 min in the dark before the fluorescence was recorded.

The fluorescence spectrum in the presence of ligand was subtracted from DMF baseline obtained mixing the protein with the same amounts of carrier without ligand. Binding was assayed with RA 5 μM (1:10 protein:ligand concentration) and a DMF final concentration of 0.005%.

### Image acquisition and analysis

Labeled cells were visualized with an Eclipse 90i fluorescence microscope (Nikon) equipped with a DS-Ri1 (Nikon) digital CCD camera. Images were acquired under the same conditions of illumination, diaphragm and condenser adjustments, exposure time, background correction and color levels.

Confocal images were obtained with a 63x oil immersion objective (HCX PL Apo CS NA = 1.4; Leica) attached to a confocal DMI 6000B microscope with a TCS SP5 confocal system (Leica) equipped with AOBS and AOTF systems. Fluorophores were excited with WLL laser (Leica) and a 405 line (Leica) controlled by LAS AF software (Leica). Emissions were collected with the AOBS system and three spectral detectors. Laser power and detection gains were set by scanning control samples labeled with secondary antibody alone. We ensured to obtain similar dynamic ranges in our images, and adjusted gain and offset using LUTs. In this manner, bleedthrough can be neglected. Negative control images showed very weak and homogeneous background. We obtained confocal sections under constant conditions to minimize image acquisition variation. Images were stored as 1024x1024 pixels and 8-bit TIFF files.

Z-series (xyz scan) were performed. The number of z-stacks was determined by observing the limits of the cell membrane. The focus plane was set to be 3 μm beneath the section surface. The optimal value of the step size was calculated for the wavelength used to fulfill the Nyquist theorem. The optical section thickness was 0.772 μm. Besides, images were taken with a 4x zoom, reducing field size. Pixel size corresponded to 0.06*0.06*0.3777 μm^3^. Scanning was performed with a 1.0 Airy unit pinhole size.

Images were processed with a Gaussian Blur filter [Sigma (Radius): 1.00], to facilitate object detection, and analyzed with the Colocalization Indices plug-in [50] and the 3D Object Counter tool using FIJI software. To analyze triple-colocalization experiments we used the Image Calculator and 3D Object Counter tools of FIJI.

A principal component analysis (PCA) was performed on the 54 different variables per cell retrieved from our image analysis to reduce its dimensionality. Nine components were found with informative value, and three of them explained over 55% of the data variability. Intensity correlation quotient (ICQ) [49] variables were heavily represented in the first component. Pixel overlap proportions, and number and volume of objects were also of interest. Thus, we run a second PCA with 11 variables (S1A Fig). ICQ (variables 1–3) and Pixel Overlap (variable 4) presented the largest weight in component 1 (accounting for 31.5% of variability), and relative Overlaps (variables 5–7) were the most important for component 2 (explaining 21.3% of variability). The first component score of this PCA was used to assess for global statistical differences due to the experimental conditions (S1B Fig). Only EEA1 and Lamp2 showed significant variation between control and PQ condition (Two-way ANOVA, Holm-Sidak post-hoc method, p < 0.05).

This multivariate analysis helped us to focus on two main colocalization variables relative to ApoD signal (1 and 5; arrows in S1A Fig) and the number and volume of ApoD-positive objects (variables 8 and 11) to understand the dynamics of ApoD traffic in the cell. It also helps us to focus on the PQ-dependent changes, since most variables covariate between control and LS conditions in our two-way ANOVA study.

### Lysosomal pH measurement

Lysosomal pH was measured using the dye LysoSensor Yellow/Blue DND-160 (Life Technologies) as described [71,72,73,74]. As a ratiometric dye, the LysoSensor readout is independent of concentration. Moreover, because it is membrane permeable, its readout is representative of a broad range of lysosomes in comparison with a dextran-tagged probe that reach lysosomes by endocytosis. Experimental parameters such as incubation time and dye concentration have been set to minimize variation and to give the best signal-to-noise ratio [74].

### LysoSensor ratiometric excitation analysis for lysosomal pH in cell populations

Cells were grown to >80% confluence in black 96-well plates (NUNC), plated in alternating rows to control for any signal variation across the plate. After removal of medium and washes with PBS, cells were incubated for 3 min with 2 μM LysoSensor Yellow/Blue in isotonic solution (NaCl, 105 mM; KCl, 5 mM; HEPES-Acid, 6 mM; Na-HEPES, 4 mM; NaHCO_3_, 5 mM; mannitol, 60 mM; glucose, 5 mM; MgCl_2_, 0.5 mM; CaCl_2_, 1.3 mM; pH adjusted to 7.4). Dye loading and incubation steps were carried out at room temperature in the dark. After 3 min, cells were rinsed three times in isotonic solution, and incubated with either additional isotonic solution or with pH calibration buffers. After 10 min fluorescence was measured with a GENios Pro Fluorometer and recorded using the XFluor4GENiosPro software package (TECAN). Lysosomal pH was determined from the ratio of excitation light at 340 nm and 390 nm (F340 nm/F390 nm, 535 nm emission) [16]. Mean light levels at both excitation wavelengths were integrated over 2000 μs and recorded for each well. This step was repeated after 5 ms for each sample. The final calculated pH represents the mean of six measurements taken strictly 12 min after dye removal, as LysoSensor generates lysosomal alkalinization with longer incubation times.

Absolute pH levels were obtained by calibrating lysosomal pH against standards. Cells present in calibration wells were incubated with 15 μM monensin and 30 μM nigericin (SIGMA), proton-cationophores that permeabilize the lysosomal membrane to Na^+^ and K^+^, respectively. These ionophores were added in a solution of 20 mM MES (2-(N-Morpholino)ethanesulfonic acid), 110 mM KCl and 20 mM NaCl, with pH 4.0, 4.5, 5.0, 5.5 and 6.0, forcing lysosomes to equilibrate with those pH values (S5A Fig).

### LysoSensor confocal emission spectra analysis for single lysosomal pH combined with ApoD immunocytochemistry

For LysoSensor staining, cells attached to poly-L-lysine coverslips were washed in warm isotonic solution, and incubated with isotonic solution or pH calibration buffers. Confocal fluorescence images were then obtained by exciting at 405 nm, and the emission collected at 420–700 nm (λ scan (xyz)) taking two averaged images every 10 nm.

Using the LAS AF Lite software (LEICA), we created ROIs for each lysosome and their emission spectra were obtained. Each spectrum was fitted to a five parameters Weibull’s equation (S5B and S5D Fig). The emission 470/524 nm ratio was then calculated for each sample. The lysosomal pH values were determined from the standard curve generated with the pH calibration samples (S5C Fig). Before cells were fixed and permeabilized a white field image was taken in the confocal microscope (S5D Fig step 4). Immunocytochemistry was then performed to detect ApoD in lysosomes, and each cell was position-identified to overlap ApoD to LysoSensor signals (S5D Fig steps 5–7). Images were analyzed with FIJI, and estimate the proportion of ApoD-positive and negative lysosomes we used the Image Calculator tool.

### Cathepsin B activity assay

Lysosomal Cathepsin B activity was measured using the Magic Red assay as directed by manufacturer (InmmunoChemistry Technology, LLC). Briefly, cells are exposed *in vivo* to a membrane-permeable non-fluorescent substrate that yields a red-fluorescent product upon enzymatic cleavage. Both, confocal microscopy (590 nm excitation / 630–640 nm emission) and cell population analysis in 96-well plates (570 nm excitation / 612 nm emission) were performed. L-leucyl-L-leucine methyl ester (Cayman Chemical), 2.5 mM, was used as positive control for lysosomal membrane rupture.

### Electron microscopy methods

1321N1 cells, under control or PQ conditions, destined for pre-embedding immunogold labeling of ApoD were fixed in 4% formaldehyde and 0.3% glutaraldehyde in 0.1 M phosphate buffer (PB) pH 7.4 for 30 min at 4°C. Following washes in 0.1 M PB, the cells were blocked with 0.1% cold water fish skin gelatin and permeabilized with Tween-20 (0.5%) in Tris-buffered saline (TBS; 20 mM Tris-HCL, 150 mM NaCl). Cells were incubated for 48h at 4°C with rabbit serum anti-human ApoD primary antibody [custom made by Abyntek Biopharma against purified ApoD [39]] diluted 1:500 in TBS containing Tween-20 (0.5%) and 0.1% cold water fish skin gelatin. Cells were later washed several times and incubated with ultra-small gold-conjugated goat anti-rabbit secondary antibodies (EMS, Electron Microscopy Sciences) diluted 1:50 in PBS for 48h at 4°C. After several washes with PBS, cells were post-fixed in 2% glutaraldehyde in PBS for 20 min, washed and the ultra-small gold particles were silver enhanced for 20 min at room temperature with AURION R-Gent SE-EM (Silver Enhancement for Electron Microscopy) (EMS, Electron Microscopy Sciences) following manufacturer’s indications. Later, cells were post-fixed with 0.5% OsO4 in PBS for 20 min at 4°C and washed with PBS. Cells were then dehydrated through a graded series of ethanol, and embedded in Epoxy EMbed-812 resin (EMS, Electron Microscopy Sciences). Ultrathin sections were obtained with an Ultracut E ultramicrotome (Reichert/Leica), contrasted with uranyl acetate and lead citrate, and analyzed using a JEOL JEM-1011 HR electron microscope with a CCD Gatan ES1000W camera with iTEM software.

### Myelin endocytosis and degradation assay

Myelin isolation and labeling were previously described [32].

Fluorescence microscopy was used to visualize myelin uptake by astrocytes. For this purpose, 2.5×10^5^ cells were cultured on 12 mm coverslips at 37°C in 5% CO_2_. Cells were incubated with 5 μg of DiI-labeled myelin (10 μg/ml) for three days. After incubation, non-endocytosed and unbound myelin was removed by washing twice with PBS, and cells were grown at 37°C for 2 or 6 days.

Cells were fixed in 4% formaldehyde in PBS, washed in PBS and mounted with EverBrite Mounting Medium with DAPI. DiI-labeled particles were visualized with a fluorescence microscope as described above. Number and size of myelin particles ingested by cultured astrocytes were measured from thresholded images with FIJI.

### Fly lines and maintenance

Flies were grown in a temperature-controlled incubator at 25°C, 60% relative humidity, under a 12 h light—dark cycle. They were fed on wet yeast 84 g/l, NaCl 3.3 g/l, agar 10 g/l, wheat flour 42 g/l, apple juice 167 ml/l, and propionic acid 5 ml/l. Fly females were used in all experiments. We used the line gmr:GAL4 to drive transgenes expression to the eye photoreceptors. UAS:hATXN1^82Q^ was used to trigger the neurodegenerative phenotype [75], combined with UAS:GLaz [37] and/or UAS:Dor-RNAi [48]. Recombination of two of the elements present in the second chromosome (gmr:GAL4 and UAS:GLaz) was required to obtain some of the experimental combinations. Two independent lines were used.

### Drosophila eye external morphology

Flies were anesthetized with CO_2_ and immobilized with adhesive tape. Fly eyes were photographed with a Nikon DS-L1 digital camera, in a Nikon SMZ1000 stereomicroscope equipped with a Plan Apo 1× WD70 objective. Local intensity maxima were obtained with the FIJI program, and nearest neighbor distances were calculated for each ommatidium. A regularity index (IREG) was estimated as described [47], and a percent recovery was calculated considering 0% the average degenerated eye and 100% the control wild type eye. Samples of 20–35 flies (3 days old) per condition and genotype were used to calculate the mean±SEM regularity index. We have used the freely available FIJI plug-in FLEYE for automated analysis of fly eye pictures [47].

### Ethics statement

Experimental procedures applied to mice were approved by the University of Valladolid Animal Care and Use Committee, and followed the regulations of the Care and the Use of Mammals in Research (European Commission Directive 86/609/CEE, Spanish Royal Decree ECC/566/2015).

### Statistical analysis

Statistical analyses were performed with SPSS v.19 (IBM) and SigmaPlot v.11.0 (Systat) softwares. A *p* value < 0.05 was used as a threshold for significant changes. The tests used for each experiment are stated in figure legends.

## Supporting information

S1 Fig A multivariate analysis of ApoD/organelle markers fluorescent signals guide the selection of the most explicative variables. **A.** Principal component analysis (PCA) of 11 variables selected after a first round of PCA on 54 variables per cell retrieved from our confocal z stacks image analysis. The position of a variable relative to axes (dashed lines) indicates its contribution to the two most explicative components. ICQ variables (1–3, red) show a strong weight on the first component, whereas overlap variables (4–7, green) contribute mainly to the second one. None of the object-related variables (8–11, blue) show a big impact on these components, but ApoD objects (variables 8 and 11) are more informative than the marker ones (9 and 10). Arrows point to the co-localization variables referenced to ApoD signal. **B.** Scatter plot of the image analysis datasets against the first two principal components shown in panel A. A homogeneous distribution of Caveolin and LC3 data show the lack of differences between conditions. A significant segregation of EEA1 and Lamp-2 datasets appear between control and PQ conditions (Two-way ANOVA, Holm-Sidak post-hoc method, p < 0.05).
(TIF)

S2 Fig ApoD is specifically enriched in a subset of organelles upon stress. **A-E.** Colocalization of ApoD in control and 24h PQ conditions in 1321N1 cells. Colocalization with caveola (Caveolin 1) (A), Clathrin-coated pits and vesicles (B), early endosome compartment (EEA-1) (C), late endosome-lysosome compartment (Lamp-2) (D), and autophagosomes or autophagolysosomes (LC3) (E). Representative sections of confocal microscopy z-stacks are shown. **F-G.** No colocalization was detected for ApoD with mitochondria (F) or peroxisomes (Catalase) (G). All markers were detected by immunocytochemistry except for the mitochondria, where cells were transfected with an organelle-directed GFP construct (using COX VIII signal sequence, see Methods). Colocalization appears in yellow. Calibration bars: 5 μm.
(TIF)

S3 Fig ApoD is a secreted protein and uses canonical synthesis and secretion pathways. **A.** Immunoblot analysis of native hApoD expressed by 1321N1 astroglial cells, detected in both cell extracts (arrow) and concentrated (20x) culture medium (asterisk). **B.** Time course of ApoD accumulation in the culture medium of HEK293T cells transfected with a hApoD expression plasmid (no concentration of media required). **C.** Representative confocal microscopy section of a 1321N1 cell transfected with a RER-targeted GFP expression plasmid (using the calreticulin signal sequence). ApoD is detected by immunocytochemistry. **D.** Colocalization of hApoD with RER in HEK293T cells cotransfected with RER-targeted GFP construct and hApoD plasmid, see Methods). Calibration bars: 5 μm.
(TIF)

S4 Fig Autophagosomes distribution in response to oxidative stress. **A.** Representative images of immunocytochemical localization of LC3 in 1321N1 astroglial cells at 2 and 24 h of PQ treatment. Calibration bars: 5 μm. **B.** Number and volume of LC3-positive objects in control and after 2 or 24 h PQ treatment. LC3-positive autophagosomes increase in size and decrease in number along oxidative stress treatment, revealing autophagy flow. Error bars represent SEM (n = 20 cells/condition from two independent experiments). Object volume was measured by number of pixels/voxel. Statistical differences were assessed by ANOVA on Ranks (p<0.001) with Tukey post-hoc method (p<0.05, denoted by asterisks).
(TIF)

S5 Fig LysoSensor fluorescence spectra analysis for pH measurements in cell populations, and in single lysosomes combined with ApoD immunolabeling. **A.** Calibration curves obtained from excitation spectra (ratio 340 nm/380 nm) for the cell types used in this work after forcing lysosomal pH to equilibrate with known extracellular pH (see Methods). **B.** Representative fluorescence emission spectra of single lysosomes in confocal sections, fitted to a five-parameter Weibull function, after equilibrating lysosomal pH to different extracellular pH. Dashed lines point to the pH values (470 nm/524 nm) selected to calculate the ratio. **C.** Calibration curve for 1321N1 cells confocal emission spectra from single lysosomes. **D.** Schematic representation of the protocol devised to measure single lysosome pH combined with ApoD labeling. Steps: 1) *In vivo* imaging; 2) Selection of region of interest (ROI); 3) LysoSensor spectra analysis and non-linear regression fitting; 4) White field image before cell fixation; 5) Native ApoD immunodetection; 6) Cell identification (guided by bright-field image); 7) Selection of ApoD positive/negative lysosomes for analysis. Calibration bars: 10 μm.
(TIF)
