# Supplementary figures and images for: Protecting cells by protecting their vulnerable lysosomes: Identification of a new mechanism for preserving lysosomal functional integrity upon oxidative stress
Source: PLoS Genet. 2017 Feb 9;13(2):e1006603. doi: 10.1371/journal.pgen.1006603 (PMC5325589; doi:10.1371/journal.pgen.1006603)

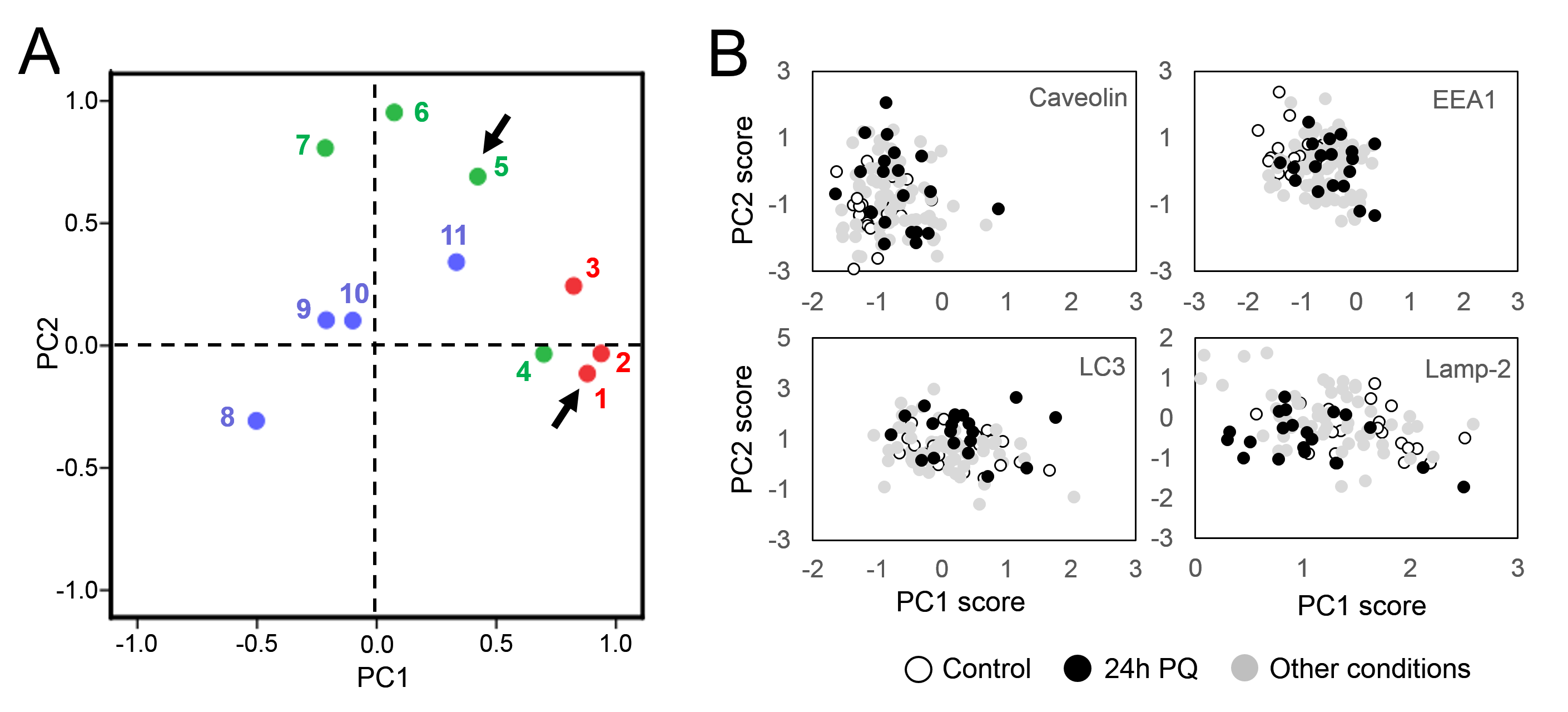

Supplement: S1 Fig — A. Principal component analysis (PCA) of 11 variables selected after a first round of PCA on 54 variables per cell retrieved from our confocal z stacks image analysis. The position of a variable relative to axes (dashed lines) indicates its contribution to the two most explicative components. ICQ variables (1–3, red) show a strong weight on the first component, whereas overlap variables (4–7, green) contribute mainly to the second one. None of the object-related variables (8–11, blue) show a big impact on these components, but ApoD objects (variables 8 and 11) are more informative than the marker ones (9 and 10). Arrows point to the co-localization variables referenced to ApoD signal. B. Scatter plot of the image analysis datasets against the first two principal components shown in panel A. A homogeneous distribution of Caveolin and LC3 data show the lack of differences between conditions. A significant segregation of EEA1 and Lamp-2 datasets appear between control and PQ conditions (Two-way ANOVA, Holm-Sidak post-hoc method, p < 0.05). (TIF) [file pgen.1006603.s001.tif]

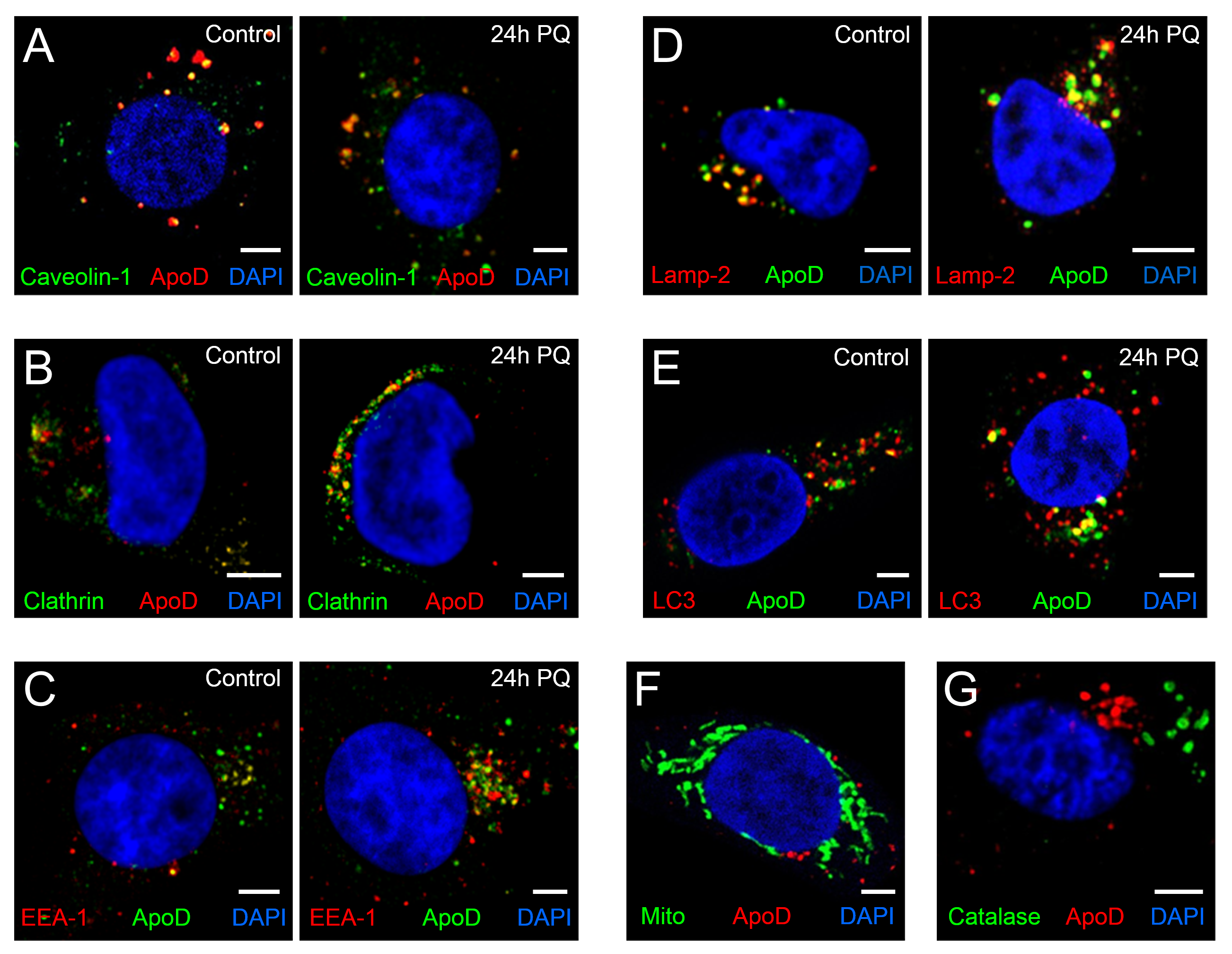

Supplement: S2 Fig — A-E. Colocalization of ApoD in control and 24h PQ conditions in 1321N1 cells. Colocalization with caveola (Caveolin 1) (A), Clathrin-coated pits and vesicles (B), early endosome compartment (EEA-1) (C), late endosome-lysosome compartment (Lamp-2) (D), and autophagosomes or autophagolysosomes (LC3) (E). Representative sections of confocal microscopy z-stacks are shown. F-G. No colocalization was detected for ApoD with mitochondria (F) or peroxisomes (Catalase) (G). All markers were detected by immunocytochemistry except for the mitochondria, where cells were transfected with an organelle-directed GFP construct (using COX VIII signal sequence, see Methods). Colocalization appears in yellow. Calibration bars: 5 μm. (TIF) [file pgen.1006603.s002.tif]

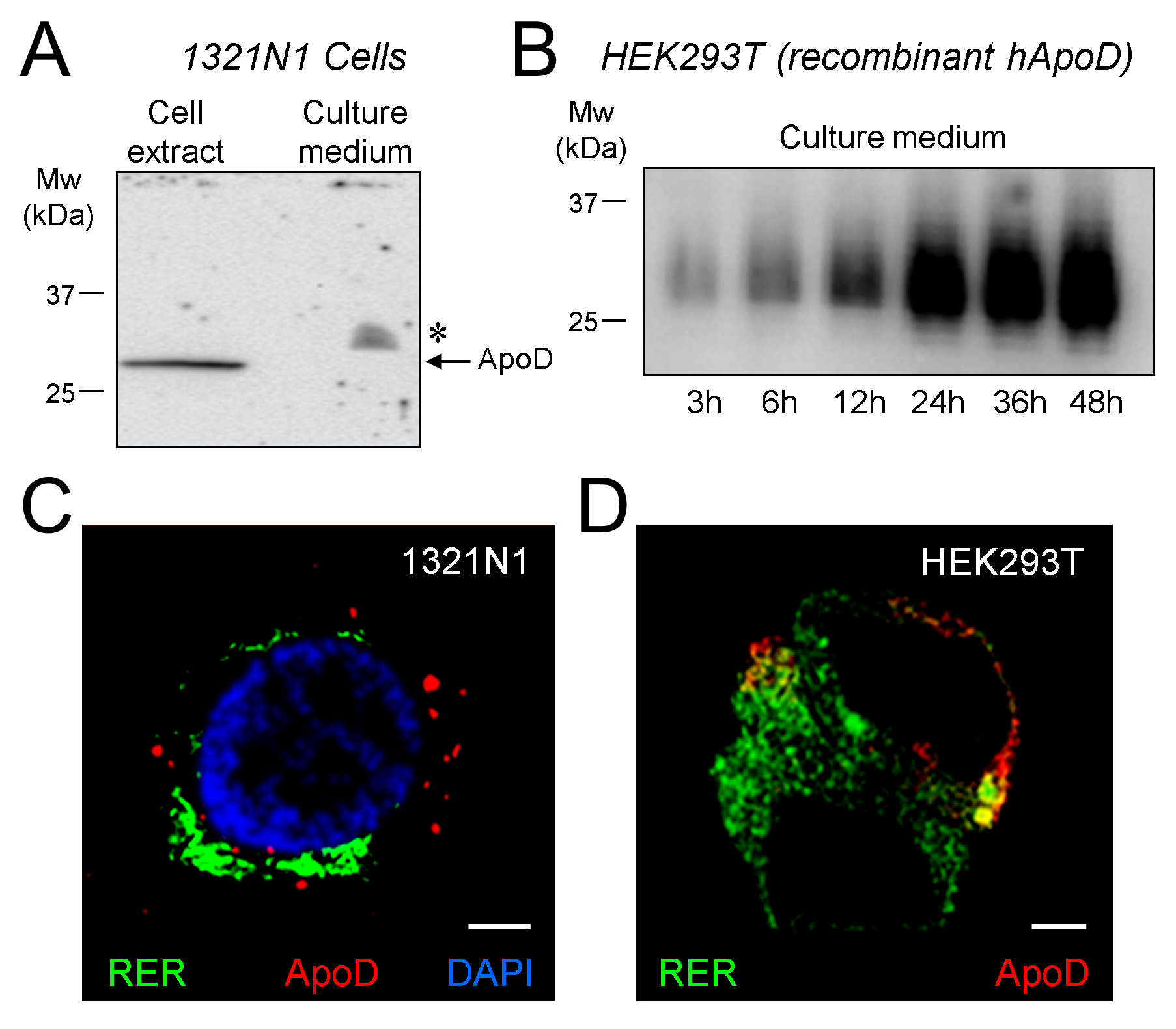

Supplement: S3 Fig — A. Immunoblot analysis of native hApoD expressed by 1321N1 astroglial cells, detected in both cell extracts (arrow) and concentrated (20x) culture medium (asterisk). B. Time course of ApoD accumulation in the culture medium of HEK293T cells transfected with a hApoD expression plasmid (no concentration of media required). C. Representative confocal microscopy section of a 1321N1 cell transfected with a RER-targeted GFP expression plasmid (using the calreticulin signal sequence). ApoD is detected by immunocytochemistry. D. Colocalization of hApoD with RER in HEK293T cells cotransfected with RER-targeted GFP construct and hApoD plasmid, see Methods). Calibration bars: 5 μm. (TIF) [file pgen.1006603.s003.tif]

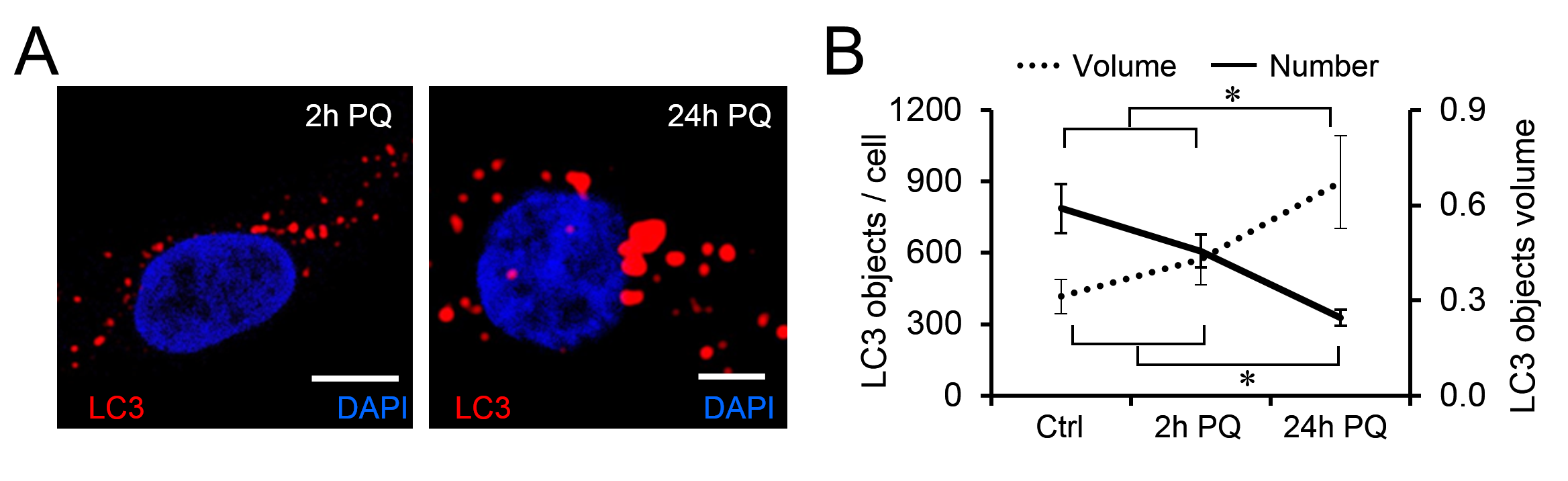

Supplement: S4 Fig — A. Representative images of immunocytochemical localization of LC3 in 1321N1 astroglial cells at 2 and 24 h of PQ treatment. Calibration bars: 5 μm. B. Number and volume of LC3-positive objects in control and after 2 or 24 h PQ treatment. LC3-positive autophagosomes increase in size and decrease in number along oxidative stress treatment, revealing autophagy flow. Error bars represent SEM (n = 20 cells/condition from two independent experiments). Object volume was measured by number of pixels/voxel. Statistical differences were assessed by ANOVA on Ranks (p<0.001) with Tukey post-hoc method (p<0.05, denoted by asterisks). (TIF) [file pgen.1006603.s004.tif]

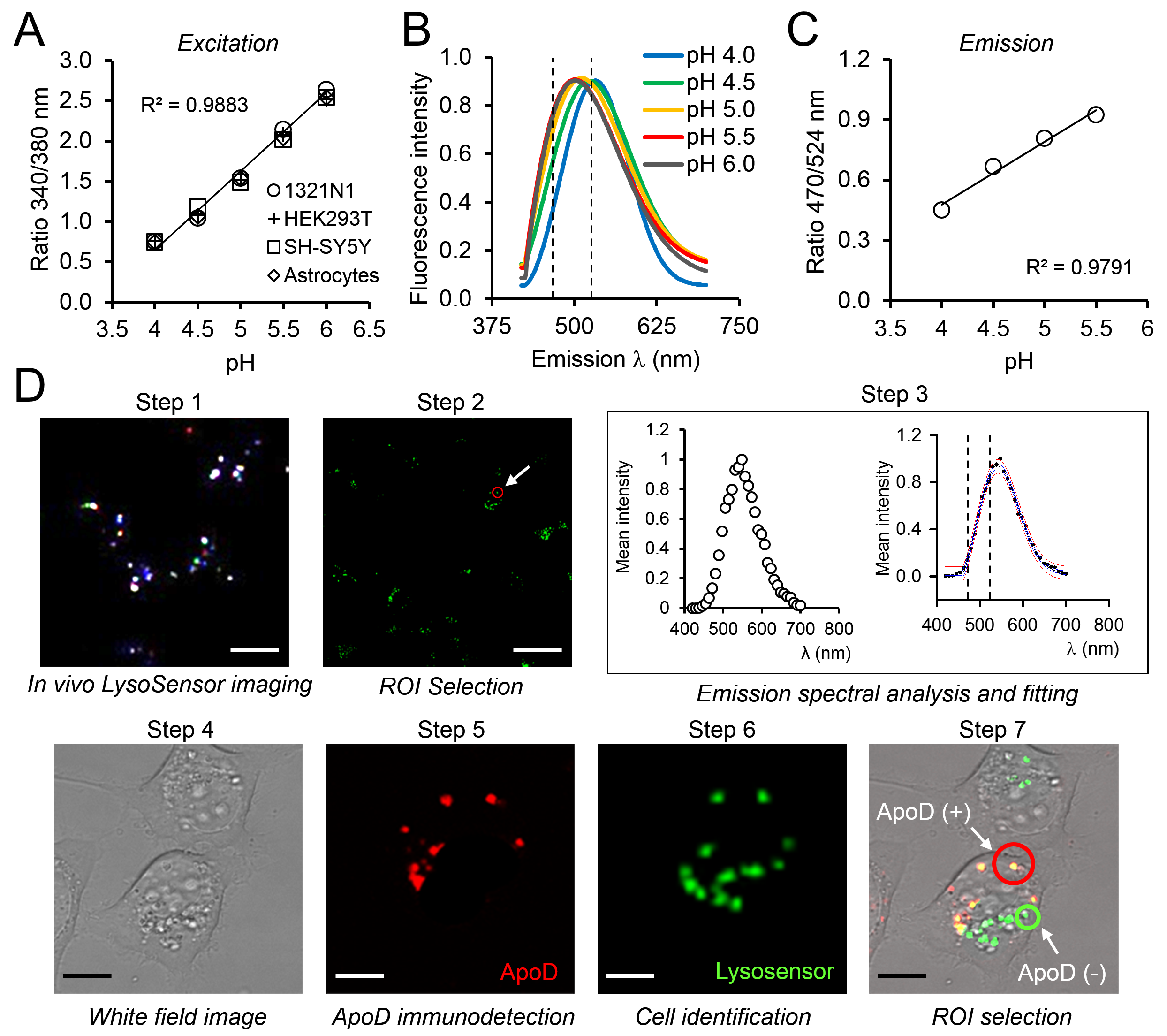

Supplement: S5 Fig — A. Calibration curves obtained from excitation spectra (ratio 340 nm/380 nm) for the cell types used in this work after forcing lysosomal pH to equilibrate with known extracellular pH (see Methods). B. Representative fluorescence emission spectra of single lysosomes in confocal sections, fitted to a five-parameter Weibull function, after equilibrating lysosomal pH to different extracellular pH. Dashed lines point to the pH values (470 nm/524 nm) selected to calculate the ratio. C. Calibration curve for 1321N1 cells confocal emission spectra from single lysosomes. D. Schematic representation of the protocol devised to measure single lysosome pH combined with ApoD labeling. Steps: 1) In vivo imaging; 2) Selection of region of interest (ROI); 3) LysoSensor spectra analysis and non-linear regression fitting; 4) White field image before cell fixation; 5) Native ApoD immunodetection; 6) Cell identification (guided by bright-field image); 7) Selection of ApoD positive/negative lysosomes for analysis. Calibration bars: 10 μm. (TIF) [file pgen.1006603.s005.tif]
